# Comparative transcriptome analyses and CRISPR/Cas9-mediated functional study of *Tfsdh1* reveal insights into the interaction between *Tremella fuciformis* and *Annulohypoxylon stygium*

**DOI:** 10.3389/fmicb.2025.1723122

**Published:** 2026-01-12

**Authors:** Yuanyuan Wang, Danyun Xu, Mei Hao, Aimin Ma

**Affiliations:** 1 College of Food Science and Technology, Wuhan Business University, Wuhan, Hubei, China; 2 College of Food Science and Technology, Huazhong Agricultural University, Wuhan, Hubei, China; 3 Hubei Engineering Research Center for Protection and Utilization of Special Biological Resources in the Hanjiang River Basin, College of Life Science, Jianghan University, Wuhan, Hubei, China

**Keywords:** *Tremella fuciformis*, *Annulohypoxylon stygium*, interaction, RNA-seq, CRISPR, sorbitol dehydrogenase

## Abstract

*Tremella fuciformis*, a famous edible and medicinal fungus, completes its life cycle in nature with the companion fungus *Annulohypoxylon stygium*. Although previous studies have initially explored the molecular mechanisms underlying this interaction, related pathways and genes in *T. fuciformis* remain poorly characterized. To address this, substrate-cultured samples were collected for RNA-seq. Differentially expressed genes (DEGs) in both *T. fuciformis* and *A. stygium* were identified and subjected to GO and KEGG annotation and enrichment analyses. Upregulated pathways were examined and DEGs associated with pentose metabolism were selected for pathway construction. One significantly upregulated gene, *Tfsdh1* (gene_sp10002100.1), was chosen for further functional validation using CRISPR/Cas9 gene editing system, a method established in our laboratory. The results revealed that the DEGs are primarily involved in carbohydrate and amino acid metabolism. Upregulated pathways were related to carbon source metabolism and stress defense, demonstrating their importance in the fungal interaction. Putative pentose catabolic pathway and oxido-reductive pathway were constructed by integrating RNA-seq data with existing literature. Phenotypic analysis demonstrated that deletion of *Tfsdh1* in *T. fuciformis* adversely affected mycelial growth rate, morphology, sorbitol utilization, SDH activity, and interaction with *A. stygium*. In conclusion, comparative transcriptome analyses provide novel insights to investigate the interaction between *T. fuciformis* and *A. stygium*. Functional research revealed that *Tfsdh1* plays a critical role in sorbitol metabolism during the interaction, providing a foundation for further elucidating the molecular mechanisms of the interaction between these two fungi.

## 1 Introduction

*Tremella fuciformis*, a famous edible and medicinal mushroom in Asia, contains substantial proteins, amino acids, and dietary fiber alongside bioactive polysaccharides, polyphenols, and terpenoids (Zhang and Wang, 2016). It exhibits significant health benefits including anti-inflammatory (Khan et al., 2022), antioxidant (Lee et al., 2023), antitumor (Xie et al., 2023), hypoglycemic (Chiu et al., 2022), and hypolipidemic effects (Li et al., 2023), as well as immunomodulatory (Xie et al., 2022) and gut microbiota regulatory activities (Wu et al., 2022). As the largest country in the production, consumption, and export of *T. fuciformis*, China faces biological challenges in sustaining this industry. Unlike most independently cultivatable mushrooms, *T. fuciformis* requires the company of *Annulohypoxylon* sp. to complete its life cycle in both natural and cultivated environments (Chen and Huang, 2002). *Annulohypoxylo*n stygium has been identified as the companion fungus of *T. fuciformis* predominantly cultivated in Fujian (Deng et al., 2016).

Research on *T. fuciformis*-*A. stygium* interaction has advanced to the molecular level. Genomic analyses reveal *A. stygium* possesses substantially more carbohydrate-active enzyme (CAZyme) genes for lignin, cellulose, and hemicellulose degradation than *T. fuciformis* (Wang, 2013; Wingfield et al., 2018; Xue et al., 2021). Transcriptome profiling of PDA co-cultures showed that the differentially expressed genes (DEGs) of *T. fuciformis* were enriched in amino acid metabolism, pentose and glucuronate interconversion, and MAPK signaling pathways, while the DEGs of *A. stygium* were clustered in secondary metabolite biosynthesis, antibiotic production, and starch and sucrose metabolism (Liu et al., 2019). Functionally, hyphal fusion forms a composite membrane through which sugar transporters of *A. stygium* facilitate small-molecule sugar being transferred to *T. fuciformis* (Lin, 2022). The alternative oxidase (AOX) of *A. stygium* enhances its antioxidant capacity while mitigating reactive oxygen species (ROS) stress in *T. fuciformis*, and inhibits the gelatinization of *T. fuciformis* hyphae through melanin regulation (Liu et al., 2022). Its tyrosinase (TYR) participates in melanin biosynthesis via the DOPA pathway and activates the DHN pathway as compensatory mechanism when the former is disrupted (Liu, 2020). In addition, catalase-peroxidase (KatG) of *T. fuciformis* contributes to ROS scavenging (Liu, 2020). However, limitations persist. Genome analyses based on the second-generation sequencing exhibit inherent accuracy constraints and co-cultures from PDA for RNA-seq inadequately reflect the real interaction in natural conditions. Crucially, functional characterization of interaction-related genes in *T. fuciformis*, particularly those involved in carbon metabolism, remains deficient, impeding the elucidation of molecular mechanisms of *T. fuciformis*-*A. stygium* interaction and the industrial advancement of *T. fuciformis*.

Sorbitol dehydrogenase (SDH), a key medium-chain dehydrogenase/reductase (MDR) member, catalyzes NAD^+^-dependent oxidation of D-sorbitol to D-fructose in eukaryotic organisms (El-Kabbani et al., 2004; Koivistoinen et al., 2012). This reaction connects polyol metabolism with glycolysis while regenerating NADH for redox homeostasis. In plants, SDH regulates the growth, development, and stress responses through sorbitol metabolism (Aguayo et al., 2013; Su et al., 2024), whereas its functionality in filamentous fungi remains poorly characterized. In *Aspergillus niger*, *sdhA* (An07g01290) is essential for D-sorbitol catabolism and participates in the oxido-reductive D-galactose pathway (ORP) (Koivistoinen et al., 2012). In *Aspergillus nidulans*, *sdhA* was highly induced by D-galactose and regulated by different regulators (GalX, GalR, AraR and XlnR), though its precise function requires validation (Meng et al., 2022). These findings indicate conserved SDH functions in fungal carbon metabolism and stress adaptation, yet its role in *T. fuciformis* during the interaction remains unexplored.

In this study, genome data from the third-generation sequencing was employed as a high-accuracy reference for transcriptome analysis of samples collected from substrate. Comparative transcriptome analysis was carried out to identify crucial pathways and candidate genes. One significantly upregulated gene (gene_sp1002100.1, the ortholog of *sdhA*, signed as *Tfsdh1*), was selected for further functional research. To validate its function in *T. fuciformis* during the interaction, the CRISPR/Cas9 genome editing system was first established to generate targeted knockout mutants of *T. fuciformis*. The growth, morphology, carbon utilization, enzyme activities, and the interaction of wild-type, mutant, and complemented strains were comparatively analyzed.

## 2 Materials and methods

### 2.1 Strains, culture media, and conditions

Two compatible monokaryotic yeast-like cells of *T. fuciformis* (*Tf*YLCs), designated Y32 and Y13, were isolated from the fruiting body of *T. fuciformis* cultured on a substrate (composition: 75 g sawdust, 20 g bran, 2 g gypsum, 1.3 g glucose, 1 g soybean meal, 0.4 g MgSO_4_·7H_2_O, 0.3 g urea, 120 g water). *A. stygium* mycelia were isolated from the same substrate mixture. Dikaryotic mycelia of *T. fuciformis*, designated Y32 × Y13, were generated by mating strains Y32 and Y13. All strains were maintained on PDA slants (potato dextrose agar; composition per liter: filtrate from 200 g boiled potatoes, 20 g dextrose, 15 g agar) at 25 °C in the laboratory of Food Microbiology, Huazhong Agricultural University. PDB (potato dextrose broth; composition per lite: filtrate from 200 g boiled potatoes, 20 g dextrose), GMB (glucose minimal broth; composition per liter: 20 g glucose, 1.32 g (NH₄)₂SO₄, 0.25 g MgSO_4_·7H_2_O, 0.5 g KH₂PO_4_, 0.2 mg VB_1_, 2 mg ZnSO_4_·7H_2_O, 0.38 g CaCl_2_, and 0.02 mg (NH_4_)_2_MoO_4_), SMB (sorbitol minimal broth; composition per liter: 20 g sorbitol, other ingredients are the same as GMB), AEB (*A. stygium* extract broth; composition per liter: water extract from 350 g *A. stygium* mycelium-substrate mixture, 5 g glucose, other ingredients are the same as GMB) and SMB (substrate extract broth; composition per liter: water extract from 350 g substrate, 5 g glucose, other ingredients are the same as GMB) were used for liquid culture of *Tf*YLCs. PDA, GMA, SMA, AEA, and SEA were prepared by adding 1.5% (w/v) agar into PDB, GMB, SMB, AEB, and SEB as required for solid culture. For transformant selection, PDA was supplemented with either hygromycin B or phleomycin at a final concentration of 50 μg/mL. All cultures were incubated at 25 °C. Y32 × Y13 dikaryotic mycelia (sample A) were cultured on PDA, a mixture of Y32 × Y13 dikaryotic mycelia and *A. stygium* mycelia (sample B) were co-cultivated on the substrate, and *A. stygium* mycelia alone (sample C) were cultivated on the substrate for 7 days, respectively. Sample A, B, and C were prepared in triplicate.

### 2.2 Transcriptome sequencing and analysis

Total RNA was extracted from Samples A, B, and C using TRIzol Reagent (Invitrogen, United States) following the manufacturer’s instructions. The concentration and purity of the extracted RNA were measured by NanoDrop 2000 microvolume spectrophotometer (Thermo Fisher Scientific, United States). RNA integrity was evaluated using an Agilent 2100 Bioanalyzer with the Agilent RNA 6000 Nano Kit. RNA sequencing libraries were prepared and subsequently sequenced on the Illumina Hiseq 2000 platform (Illumina, United States). Raw reads were processed with fastp v0.19.7 to remove adapters, unknown bases, and low-quality reads. Clean reads were aligned to the respective reference genomes of *T. fuciformis* (Accession: JBSMYS000000000) and *A. stygium* (Accession: JBSNGO000000000) using HISAT2 v2.1.0, which were sequenced by PacBio platform and assembled by our laboratory recently. The expression levels of genes were quantified as FPKM (Fragments per kilobase of transcript per million fragments mapped) values. DEGs were identified using DEseq2 v.1.4.0 with parameters: FDR <0.05, |log_2_FoldChange| ≥1. DEGs were functionally annotated through BLAST searches against the GO and KEGG databases. GO term and KEGG pathway enrichment analyses were performed using the Omicsare platform.^1^ Fungal-specific database was selected for KEGG enrichment analyses. Pathways with *q*-value <0.05 were recognized as significantly enriched pathways. Top 10 pathways in both fungi were selected based on *p*-value and the numbers of up- and down-regulated genes were normalized using the formula: (No. of upregulated genes − No. of downregulated genes)/(No. of upregulated genes + No. of downregulated genes). Bubble diagrams of top 10 pathways and network diagrams of upregulated pathways were visualized using Omicsare platform. Genes related to pentose metabolism were identified from RNA-seq data and visualized using volcano plots and heatmaps. Subsequently, putative pentose catabolic pathway (PCP) and ORP were constructed. The raw RNA-seq data are available in the NCBI SRA database under accession number PRJNA943290.

### 2.3 qRT-PCR validation

To access the accuracy of transcriptome data, 10 genes were randomly selected for qRT-PCR. Reactions were performed using SYBR Select Master Mix (Thermo Fisher Scientific, United States) on an ABI ViiA 7 Real-Time PCR System (Thermo Fisher Scientific, United States), following the manufacturer’s instructions. *β-tubulin* and *α-tubulin* were used as reference genes for *T. fuciformis* and *A. stygium*, respectively. Gene-specific primers used for qRT-PCR are listed in Supplementary Table S1. Each gene was performed with three biological replicates and three technical replicates. Relative gene expression levels were calculated using the 2^−ΔΔCT^ method. The correlation between the FPKM values and qRT-PCR data was assessed by calculating the *R*^2^.

### 2.4 Cloning and bioinformatics analyses of *Tfsdh1*

The full-length cDNA and DNA sequences of *Tfsdh1* were amplified by PCR using cDNA and genomic DNA as templates, respectively, with primers *Tfsdh1*-F/R (Supplementary Table S2). PCR products were purified and cloned into the pEASY-Blunt Zero Cloning Vector (Transgene Biotech, Beijing) for sequencing. To identify intron-exon boundaries, cDNA and DNA sequences were aligned using DNAMAN 6.0. Molecular weight and theoretical isoelectric point were predicted using ProtParam. Signal peptides were predicted via SignalP 6.0 server. For further bioinformatics analyses, sequence alignment, protein family classification, Pfam domain identification, motif discovery, and neighbor-joining phylogenetic analysis were performed using Clustal Omega, NCBI CDD, Pfam, MEME Suite, and MEGA 7.0, respectively. These analyses utilized amino acid sequences of TfSDH1 from *T. fuciformis* alongside SDH, xylitol dehydrogenase (XDH), and L-arabinitol 4-dehydrogenase (LAD) from *A. niger*, *A. nidulans*, *Aspergillus oryzae*, *Trichoderma reesei*, *Neurospora crassa* and *Saccharomyces cerevisiae*. TBtools v.2.326 was used for visualization.

### 2.5 Cloning and analysis of native *gpd* promoter

To enhance transformation efficiency, native *gpd* promoter from *T. fuciformis* was identified for plasmid construction. Primers *gpd*-chk-F/R were designed to amplify the upstream region and partial sequence of the *gpd* gene annotated in the Y32 genome (Supplementary Table S2). The Amplified fragments were cloned into the pEASY-Blunt Zero Cloning Vector (Transgene Biotech, Beijing) and subsequently sequenced. Promoter prediction was performed using Neural Network Promoter Prediction (NNPP, https://fruitfly.org/seq_tools/promoter.html) and PLANTCARE.^2^

### 2.6 Construction of knockout and complementary vectors

The backbone vector pUC19-*phleo* was first constructed, containing a phleomycin resistance cassette with the *gpd* promoter from *T. fuciformis* and the *sc3* terminator from *Schizophyllum commune*. The native *gpd* promoter was amplified from Y32 genomic DNA using primers p*gpd*-2-F/R (Supplementary Table S3). The phleomycin resistance gene and *sc3* terminator were amplified from plasmid pRO402 (kindly provided by Dr. Robin A. Ohm, Utrecht University, Netherlands) with primers *phleo*-F/R (Supplementary Table S3). These fragments were ligated into *Eco*R I (NEB, United States)-digested pUC19 using the pEASY-Uni Seamless Cloning and Assembly Kit (Transgene Biotech, Beijing).

The *Tfsdh1* deletion construct contained 1,000-bp upstream homology arm, 1,000-bp downstream homology arm, and *hph* cassette. For *hph* cassette construction, the native *gpd* promoter was amplified with primers p*gpd*-1-F/R (Supplementary Table S3), while the *hph* gene and *trpc* terminator were amplified from pSKH plasmid (kindly provided by Prof. Fusheng Chen’s laboratory, Huazhong Agricultural University, China) using primers *hph*-F/R (Supplementary Table S3). These fragments were assembled by overlap PCR and cloned into the pEASY-Blunt Zero Cloning Vector (Transgene Biotech, Beijing) to generate the intermediate construct pEASY-*hph*. The *hph* cassette was then excised from *Xho* I (NEB, United States)-digested pEASY-*hph*. The upstream and downstream homology arms of *Tfsdh1* were amplified from Y32 genomic DNA with primers *Tfsdh1*-up-1-F/R and *Tfsdh1*-down-1-F/R (Supplementary Table S3). All fragments were ligated into *Hin*d III (NEB, United States)-digested pUC19-*phleo* using the pEASY-Uni Seamless Cloning and Assembly Kit (Transgene Biotech, Beijing), yielding the *Tfsdh1* deletion vector pUC19-*Tfsdh1*-KO (Supplementary Figure S1A).

For the complementary vector pUC19-*Tfsdh1*-com (Supplementary Figure S1B), a DNA fragment containing the *Tfsdh1* gene with 1,000-bp upstream and downstream regions was amplified from Y32 genomic DNA using primers *Tfsdh1*-up-2-F and *Tfsdh1*-down-2-R (Supplementary Table S3). This fragment was cloned into *Hin*d III (NEB, United States)-digested pUC19-*phleo* using the pEASY-Basic Seamless Cloning and Assembly Kit (Transgene Biotech, Beijing).

### 2.7 Construction of knockout and complementary strains

Gene knockout and complementation in *T. fuciformis* using pre-assembled Cas9 ribonucleoproteins referred to the method established by Vonk et al. (2019) with modifications. Two candidate protospacer adjacent motif (PAM) sequences (Supplementary Figure S2) were identified in the *Tfsdh1* using CHOPCHOP.^3^ Tracr-F/R and Uni-F/R were designed for sgRNA template synthesis as previously described (Supplementary Table S4). Oligonucleotides (*Tfsdh1*-sgRNA-left-F/R and *Tfsdh1*-sgRNA-right-F/R; Supplementary Table S4) were designed and sgRNAs were synthesized *in vitro* using the T7 High Efficiency Transcription Kit (Transgene Biotech, Beijing) followed by purification with the GeneJET RNA Cleanup and Concentration Micro Kit (Thermo Fisher Scientific, United States). sgRNA concentrations were quantified using the Qubit RNA BR assay Kit (Thermo Fisher Scientific, United States). Plasmid pET-NLS-Cas9-6×His (Addgene plasmid #62934; http://n2t.net/addgene:62934; RRID:Addgene_62934; donated from David Liu) was transformed into *Escherichia coli* BL21 Star (DE3) for Cas9 expression. Cas9 purification was performed as described with modifications. Cells were lysed by ultrasonication (300 W, 3-s pulse, 5-s interval, 100 cycles). Lysates were centrifuged for 20 min at 12,000 r/min at 4 °C and supernatants were purified using Ni-NTA 6FF resin (BBI) according to the manufacturer’s protocols. For knockout strain generation, 10 μg sgRNAs, 100 μg Cas9 protein, and 100 μg pUC19 *Tfsdh1*-KO vector were co-transformed into 1 × 10^8^ CFU of Y32 prepared protoplasts. Complementary strains were generated by transforming 2–10 μg pUC19-*Tfsdh1*-com into 2 × 10^7^ CFU of *Tfsdh1* knockout protoplasts. Transformants were selected on PDA supplemented with 50 μg/mL hygromycin B and phleomycin, then screened by colony PCR using primer pairs listed in Supplementary Table S2 (Wang et al., 2019). Schematic diagram of primers for Δ*Tfsdh1* screening was shown in Supplementary Figure S7A. Putative mutants were verified by sequencing and qRT-PCR (Supplementary Table S2).

### 2.8 Phenotyping analysis

Dikaryotic strains, Y32 × Y13, Δ*Tfsdh1* × Y13 and Δ*Tfsdh1*::*Tfsdh1* × Y13, were generated by crossing Y32, Δ*Tfsdh1*and Δ*Tfsdh1*::*Tfsdh1* with Y13 on PDA, respectively. Dikaryons were purified using the hyphal tip purification technique. To test the growth of strains under different culture conditions, all strains were sub-cultured on PDA, GMA, SMA, AEA, and SEA at 25 °C for 7 days or 30 days. Y32, Δ*Tfsdh1*, and Δ*Tfsdh1*::*Tfsdh1* were cultured in PDB, GMB, SMB, AEB, and SEB at 25 °C with shaking (150 r/min). Growth in PDB was monitored at OD_600_ per 24 h for 10 days. OD_600_ of strains cultured in other liquid media was measured after 5 days. Supernatants from GMB/SMB cultures were analyzed for carbon utilization. Glucose/sorbitol content was quantified colorimetrically. SDH activity was assayed as described with modification (Teo et al., 2006). Reaction mixtures contained 100 mM Tris-HCl (pH 9.0), 1 mM NAD^+^, 300 mM sorbitol, and enzyme extract. Enzyme activity was determined by monitoring the absorbance change at 340 nm for 1 min at 25 °C. To assess the interaction between *T. fuciformis* and *A. stygium*, *A. stygium* mycelia were inoculated 1 cm away from 14-day-old *T. fuciformis* colonies on PDA plates and co-cultured at 25 °C.

### 2.9 Statistical analysis

The data was expressed as the mean ± SD. Statistical significance was performed using an independent samples *t*-test with IBM SPSS Statistics (v30). A two-tailed *p* < 0.05 was considered statistically significant.

## 3 Results

### 3.1 Transcriptome analysis

#### 3.1.1 Sequencing quality and DEG identification

**Figure 1:**
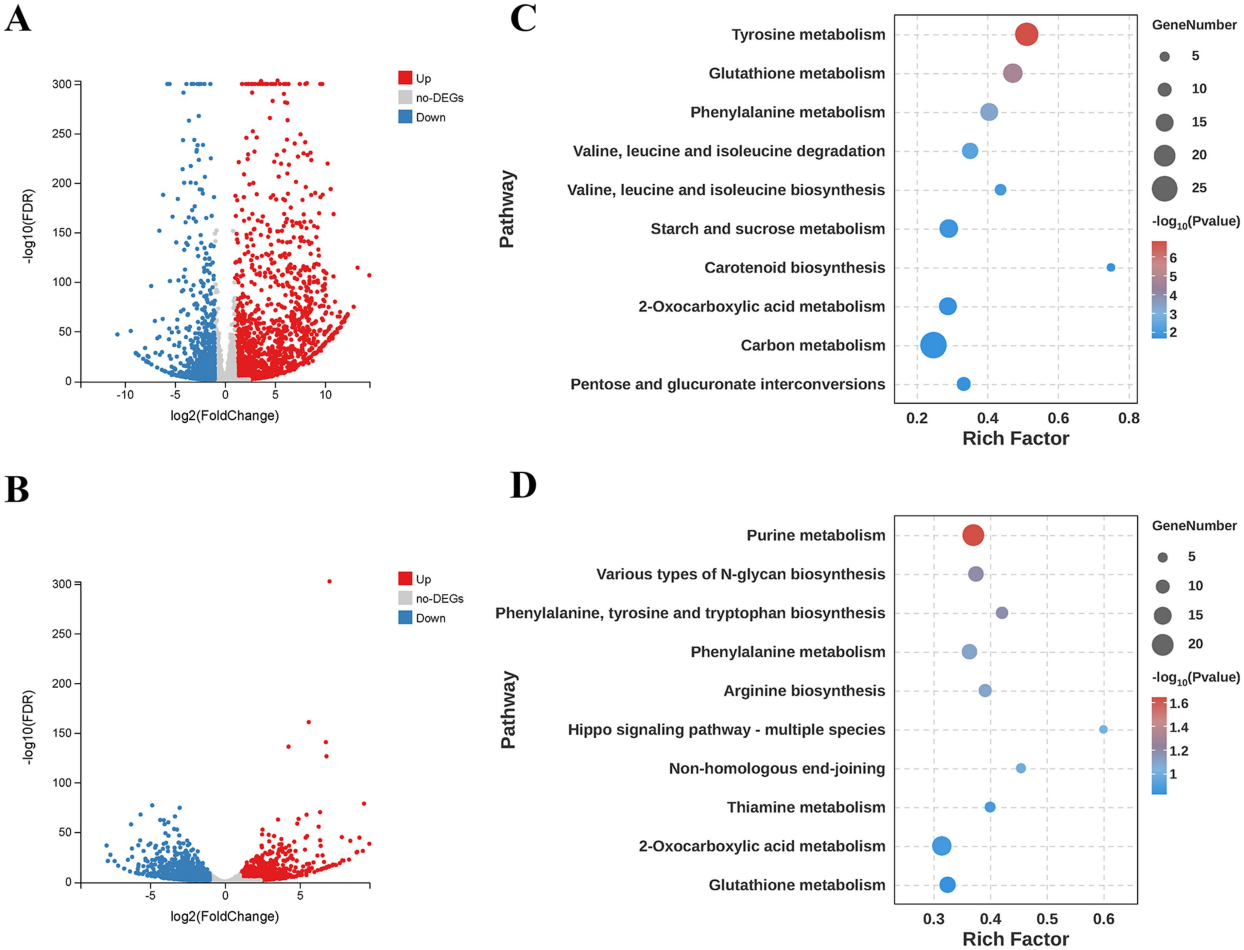
Volcano plots and KEGG enrichment analyses of DEGs from *T. fuciformis* and *A. stygium*. **(A)** Volcano plot of DEGs from *T. fuciformis*. Genes from sample A served as the control group (a), while genes of *T. fuciformis* from sample B served as the experimental group (b). DEGs of *T. fuciformis* were identified from the comparison a vs. b. **(B)** Volcano plot of DEGs from *A. stygium*. Genes from sample C served as the control group (c), while genes of *A. stygium* from sample B served as the experimental group (d). DEGs of *A. stygium* were identified from the comparison c vs. d. Red dots indicate upregulated DEGs. Gray dots indicate non-DEGs. Blue dots indicate down-regulated DEGs. FDR means false discovery rate. **(C)** Top 10 prominent KEGG pathways of DEGs from *T. fuciformis*. **(D)** Top 10 prominent KEGG pathways of DEGs from *A. stygium*. Rich factor represents the ratio of the number of DEGs mapped to a KEGG pathway to the total number of transcripts annotated to that pathway. A larger rich factor indicates a higher degree of pathway enrichment.

After data filtering and trimming, the nine cDNA libraries yielded clean reads of 60.04, 82.68, 67.83, 59.92, 56.46, 55.76, 62.20, 57.77, and 50.71 Mb, respectively, with Q20 >98% and Q30 >95% (Table 1). Clean reads from different samples were mapped to their respective reference genomes, with mapping rates ranging from 84.34 to 97.83% (Table 1). Comparative analysis identified 1,428 DEGs in *T. fuciformis* (692 upregulated, 736 downregulated; Figure 1A), and 2,113 DEGs in *A. stygium* (1,117 upregulated, 1,211 downregulated; Figure 1B) during co-culture using a threshold of |Log_2_FoldChange| ≥1 and FDR <0.05. The fold changes of these DEGs ranged from a 14.01-fold upregulation to a 12.59-fold downregulation in *T. fuciformis*, and from a 12.07-fold upregulation to a 9.88-fold downregulation in *A. stygium*. Expression patterns of 10 randomly selected DEGs were consistent with transcriptome results. During the interaction, 6 DEGs were downregulated in *T. fuciformis*, while 1 DEG was downregulated and 3 DEGs were upregulated in *A. stygium* (Supplementary Figures S3A,B). Strong correlation between datasets (*R*^2^ = 0.9602; Supplementary Figure S3C) confirmed the reliability of our transcriptome data for subsequent analyses and functional studies.

**Table 1:** Statistics of reads quality.

Sample	Raw reads (Mb)	Clean reads (Mb)	Clean reads Q20 (%)	Clean reads Q30 (%)	Clean reads ratio (%)	Mapping genome ratio (%)
A1	61.76	60.04	98.47	95.85	97.22	84.39
A2	85.17	82.68	98.35	95.55	97.08	84.34
A3	70.01	67.83	98.29	95.43	96.88	84.47
B1	62.68	59.92	98.35	95.53	95.58	89.56
B2	61.55	56.46	98.16	95.16	91.73	88.83
B3	59.06	55.76	98.22	95.28	94.4	88.97
C1	64.82	62.2	98.38	95.53	95.96	97.32
C2	60.28	57.77	98.31	95.37	95.84	97.83
C3	52.89	50.71	98.32	95.35	95.87	97.53

Q20: percentage of bases with sequencing error rate lower than 1%; Q30: percentage of bases with sequencing error rate lower than 0.1%.

#### 3.1.2 Functional annotation and enrichment of DEGs

The functional distribution of DEGs existed minor differences between *T. fuciformis* and *A. stygium*. In *T. fuciformis*, the dominant GO terms were “metabolic process” (443 DEGs), “catalytic activity” (394 DEGs), and “membrane” (295 DEGs). A notable up-regulation (226 upregulated genes, 217 down-regulated genes) was observed specifically within the “metabolic process” category. In contrast, DEGs of *A. stygium* was primarily assigned to “metabolic process” (647 DEGs), “catalytic activity” (629 DEGs), and “cell” (230 DEGs). Moreover, for both “metabolic process” (328 upregulated genes, 319 downregulated genes) and “catalytic activity” (323 upregulated genes, 306 downregulated genes) in *A. stygium*, there was a higher proportion of upregulated genes compared to down-regulated genes (Supplementary Figure S4). KEGG pathway annotation results indicated that the majority of DEGs in *T. fuciformis* and *A. stygium* were involved in metabolism pathway, among which DEGs of both fungi mainly involved in carbohydrate metabolism (75 and 73 DEGs, respectively) and amino acid metabolism (66 and 80 DEGs, respectively) (Supplementary Figure S5). KEGG pathway enrichment analysis identified significantly enriched pathways (*q*-value <0.05) in *T. fuciformis*, including tyrosine metabolism (ko00350), glutathione metabolism (ko00480), and phenylalaline metabolism (ko00360). In contrast, no pathways in *A. stygium* reached statistical significance. However, to explore biological trends, the top 10 most prominent pathways ranked by *p*-value for each species were presented in Figures 1C,D.

**Figure 2:**
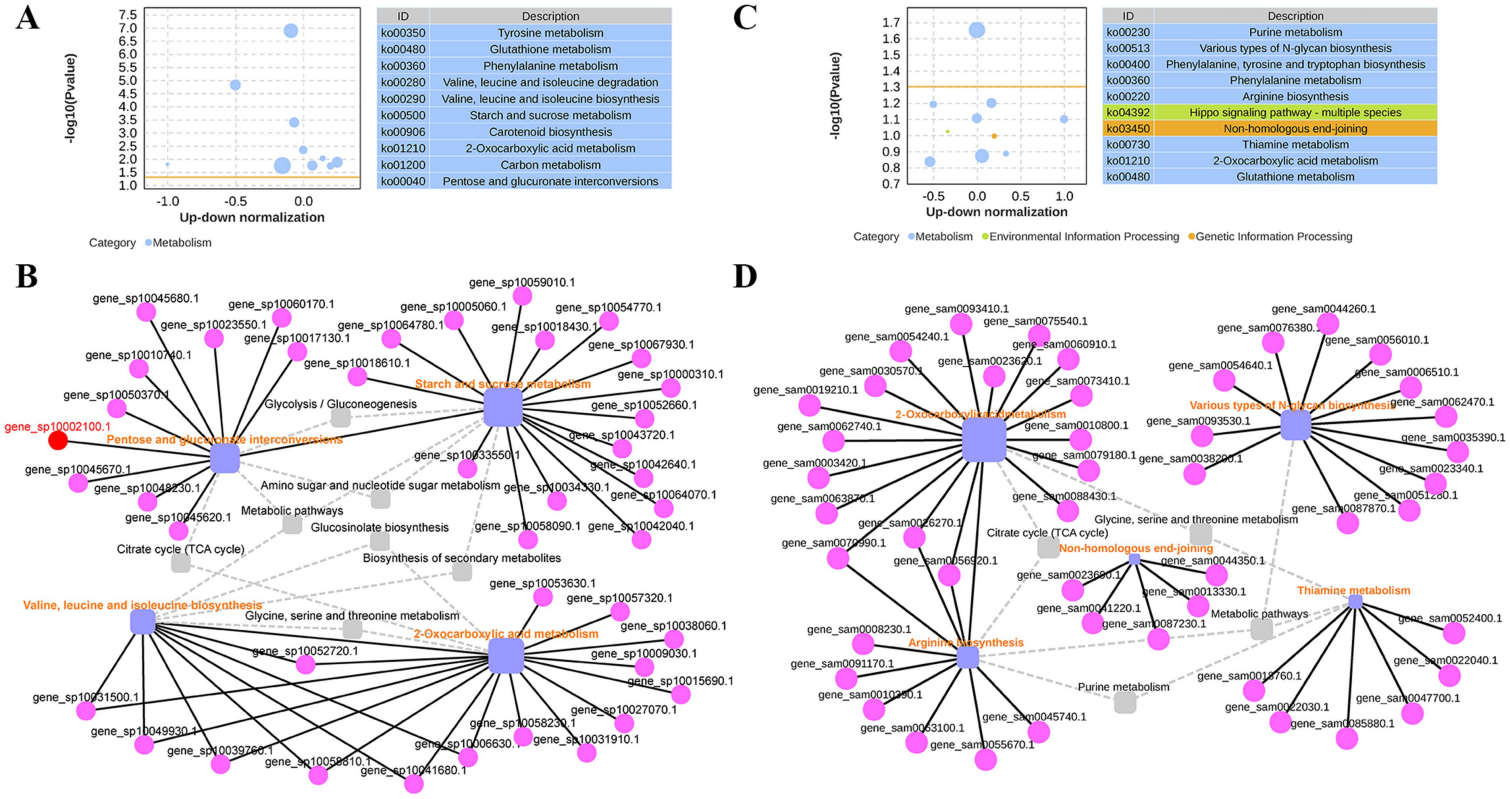
Up-down normalization analyses of top 10 prominent KEGG pathways and network analyses of upregulated pathways in *T. fuciformis* and *A. stygium*. **(A)** Up-down normalization analyses of the top 10 prominent KEGG pathways in *T. fuciformis*. **(B)** Network diagram of upregulated pathways among the top 10 prominent KEGG pathways in *T. fuciformis*. **(C)** Up-down normalization analyses of the top 10 prominent KEGG pathways in *A. stygium*. **(D)** Network diagram of upregulated pathways among the top 10 prominent KEGG pathways in *A. stygium*. The bubble size indicates the number of DEGs assigned to the current pathway. The yellow line represents the threshold for *p*-value = 0.05. On the right is a list of the top 10 pathways by *p*-value, with different colors representing different KEGG A class categories. Purple squares indicate upregulated pathways and the square size indicates the number of DEGs assigned to each pathway. Pink nodes indicate DEGs. Gray squares indicate relevant metabolic pathways. Solid lines connect genes to their assigned pathways, while dashed lines represent functional relationships between pathways, both as defined by the KEGG database.

Among the top 10 prominent pathways in *T. fuciformis*, four upregulated pathways were identified. These included valine, leucine and isoleucine biosynthesis (ko00290) with 7 DEGs (4 up, 3 down), starch and sucrose metabolism (ko00500) with 16 DEGs (10 up, 6 down), 2-oxocarboxylic acid metabolism (ko01210) with 15 DEGs (8 up, 7 down), and pentose and glucuronate interconversions (ko00040) with 10 DEGs (6 up, 4 down) (Figure 2A). Starch and sucrose metabolism directly interfaces with pentose and glucuronate interconversions, while valine, leucine, isoleucine biosynthesis converges with 2-oxocarboxylic acid metabolism. Between valine, leucine, isoleucine biosynthesis and 2-oxocarboxylic acid metabolism, 7 common DEGs are shared, representing 100% of the DEGs in the former pathway and 46.67% in the latter. These four pathways further interconnect through shared intermediary metabolic routes as defined in the KEGG database (Figure 2B).

Among the top 10 prominent pathways in *A. stygium*, five upregulated pathways were identified. These included various types of N-glycan biosynthesis (ko00513) with 12 DEGs (7 up, 5 down), arginine biosynthesis (ko00220) with 9 DEGs (9 up, 0 down), non-homologous end-joining (ko03450) with 5 DEGs (3 up, 2 down), thiamine metabolism (ko00730) with 6 DEGs (4 up, 2 down), and 2-oxocarboxylic acid metabolism (ko01210) with 17 DEGs (9 up, 8 down) (Figure 2C). Arginine biosynthesis directly intersects with 2-oxocarboxylic acid metabolism. These two pathways share 3 common DEGs, corresponding to 33.33% of the DEGs in arginine biosynthesis and 17.65% of those in 2-oxocarboxylic acid metabolism. Various types of N-glycan biosynthesis and thiamine metabolism lack direct linkages to each other and to the aforementioned pathways, instead connect through intermediate pathways. Non-homologous end-joining was identified as a relatively independent without any connections to these upregulated pathways (Figure 2D).

#### 3.1.3 Pentose catabolism in *Tremella fuciformis* and *Annulohypoxylon stygium* during interaction

**Figure 3:**
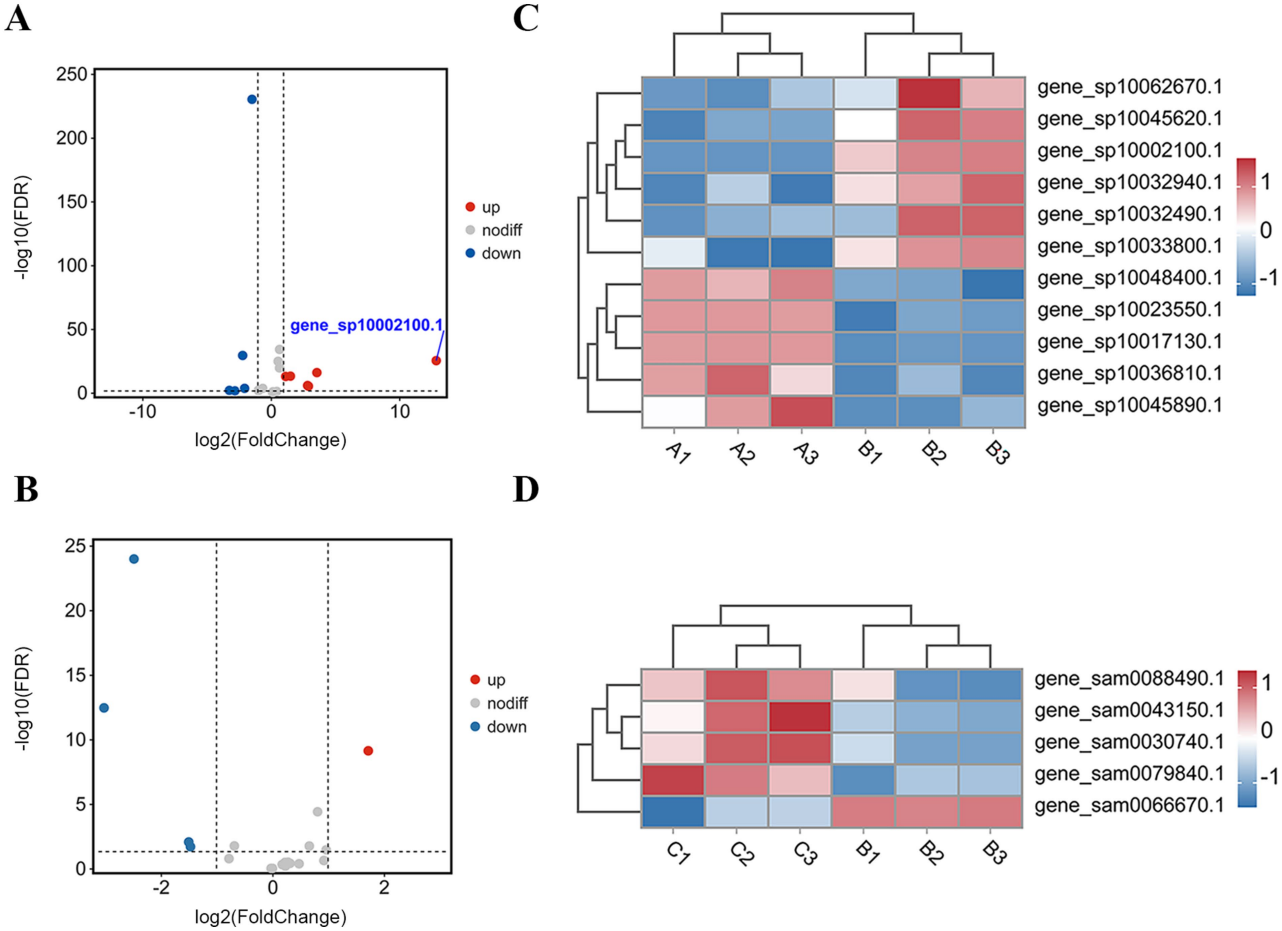
DEGs involved in pentose catabolism in *T. fuciformis* and *A. stygium*. **(A)** Volcano plots of DEGs involved in pentose catabolism in *T. fuciformis*. **(B)** Volcano plots of DEGs involved in pentose catabolism in *A. stygium*. Red dots indicate upregulated DEGs. Gray dots indicate non-DEGs. Blue dots indicate down-regulated DEGs. FDR means false discovery rate. **(C)** Cluster analysis of DEGs involved in pentose catabolism in *T. fuciformis*. **(D)** Cluster analysis of DEGs involved in pentose catabolism in *A. stygium*. Each row represents one DEG, and each column represents one sample. Blue indicates upregulated genes. Red indicates downregulated genes.

Among all DEGs in the upregulated pathways, gene_sp1002100.1 exhibited the most pronounced upregulation (log₂FC = 12.88) and was annotated to the “Pentose and glucuronate interconversions” pathway. This pathway is central to pentose catabolism and is critical for processing hemicellulose-derived pentoses, thus offering key insights into the metabolic adaptation of *T. fuciformis* during fungal interaction. We therefore selected it for further analysis. A total of 23 genes involved in pentose catabolism were identified in *T. fuciformis* during the interaction, among which 6 were upregulated, 5 were downregulated, and the remaining 12 exhibited non-differential expression (Figure 3A). In contrast, 19 pentose catabolism-related genes were detected in *A. stygium*. Only 1 was upregulated, 4 were downregulated, and 14 showed no significant expression changes (Figure 3B). The specific expression profiles of these DEGs were visualized via heatmaps (Figures 3C,D).

**Figure 4:**
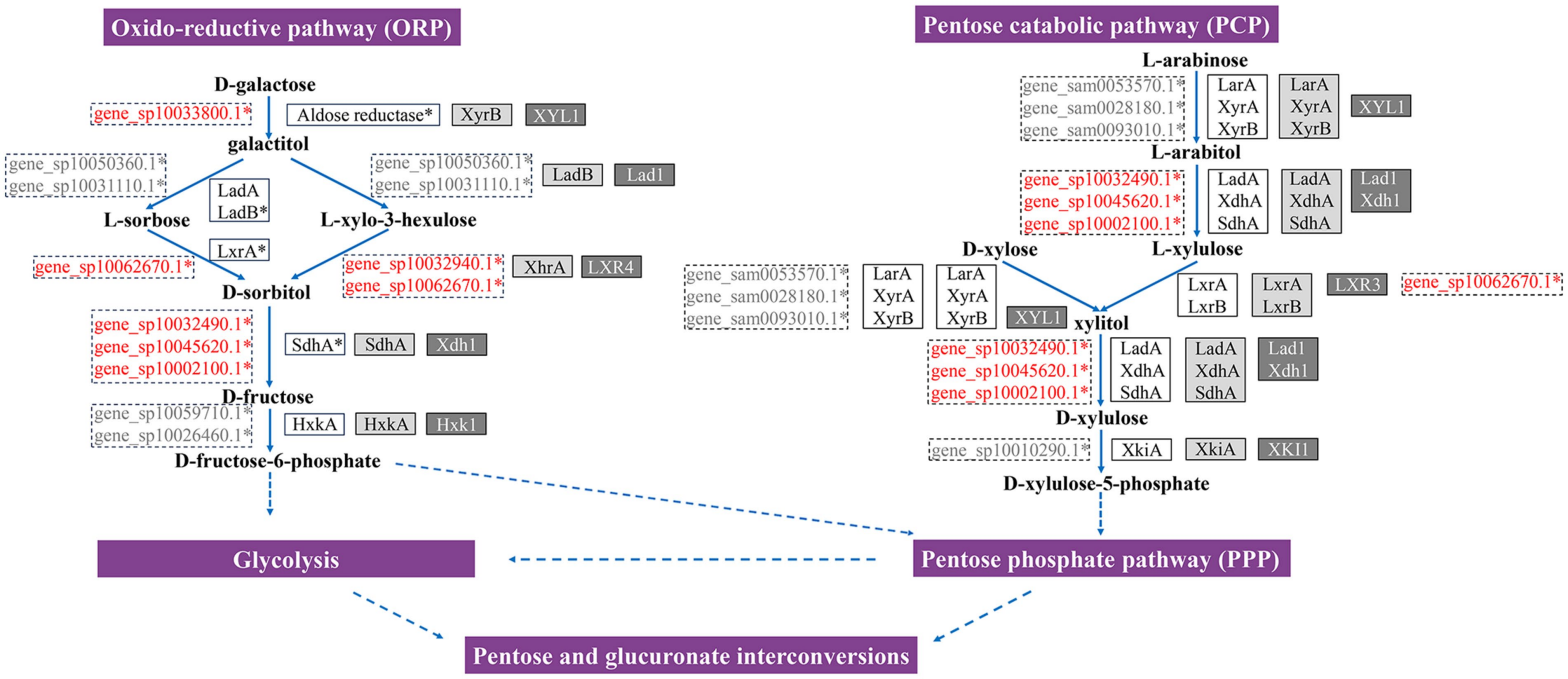
Putative oxido-reductive D-galactose pathway (ORP) and pentose catabolic pathway (PCP) in *T. fuciformis* during its interaction with *A. stygium*. The enzymes that participate in particular metabolic steps in *T. fuciformis* or *A. stygium* (dashed boxes), *A. nidulans* (white boxes), *A. niger* (light gray boxes), and *T. reesei* (dark gray boxes) are indicated. Upregulated genes are indicated in red front. Non-DEGs are indicated in gray front. LarA = L-arabinose reductase, XyrA, XyrB, and XYL1 = D-xylose reductases, LadA and Lad1 = L-arabitol dehydrogenase, LadB = galactitol dehydrogenase, XdhA and Xdh1 = xylitol dehydrogenase, SdhA = D-sorbitol dehydrogenase, LxrA, LxrB, LXR3 and LXR4 = L-xylulose reductases, XkiA and XKI1 = D-xylulose kinase, XhrA = L-xylo-3-hexulose reductase, HxkA and Hxk1 = hexokinase.

Based on the annotation and expression data of all relevant genes provided in Supplementary datasets, we proposed putative pathways for pentose catabolism and D-galactose oxidation–reduction that operate in *T. fuciformis* during its interaction with *A. stygium*. The pathway steps are supported by genes from both fungi, selected according to a hierarchical criterion: upregulated genes > non-DEGs > genomically present but not expressed, applied first in *T. fuciformis* and then to *A. stygium* (Figure 4). L-arabinose was reduced to L-arabitol by NADPH-dependent D-xylose reductase derived from *A. stygium* (gene_sam0053570.1, gene_sam0028180.1, gene_sam0093010.1). NAD^+^-dependent XDH and SDH mediate the conversion of L-arabitol to L-xylulose (gene_sp10032490.1, gene_sp10045620.1, gene_sp10002100.1), and the resulting L-xylulose is further reduced to xylitol by a NADPH-dependent L-xylulose reductase (gene_sp10062670.1). For D-Xylose metabolism, the pathway initiates with the reduction of D-xylose to xylitol by a NADPH-dependent D-xylose reductase from *A. stygium* (gene_sam0053570.1, gene_sam0028180.1, gene_sam0093010.1). Xylitol is then oxidized to D-xylulose via NAD^+^-dependent XDHand SDH (gene_sp10032490.1, gene_sp10045620.1, gene_sp10002100.1). Finally, D-xylulose is converted to D-xylulose-5-phosphate by an ATP-dependent D-xylulose kinase (gene_sp10010290.1) for entry into the pentose phosphate pathway (PPP).

In the D-galactose oxidative-reductive pathway (Figure 4), D-galactose is reduced to galactitol by aldose reductase (gene_sp10033800.1). Galactitol is subsequently catalyzed to L-sorbose or L-xylo-3-hexulose by L-arabitol dehydrogenase (gene_sp10050360.1, gene_sp10031110.1). L-sorbose is then reduced to D-sorbitol by L-xylulose reductase (gene_sp10062670.1), while L-xylo-3-hexulose is then reduced to D-sorbitol by L-xylulose reductase (gene_sp10062670.1) or L-xylo-3-hexulose reductase (gene_sp10032940.1). D-sorbitol is converted to D-fructose by either XDHor SDH (gene_sp10032490.1, gene_sp10045620.1, gene_sp10002100.1). In the final step, D-fructose is converted to D-fructose-6-phosphate by hexokinase (gene_sp10059710.1, gene_sp10026460.1), which feeds into Glycolysis. gene_sp10002100.1, signed as *Tfsdh1*, the most significantly upregulated gene in the proposed pathway, was selected for subsequent functional characterization.

### 3.2 Identification and characterization of *Tfsdh1*

**Figure 5:**
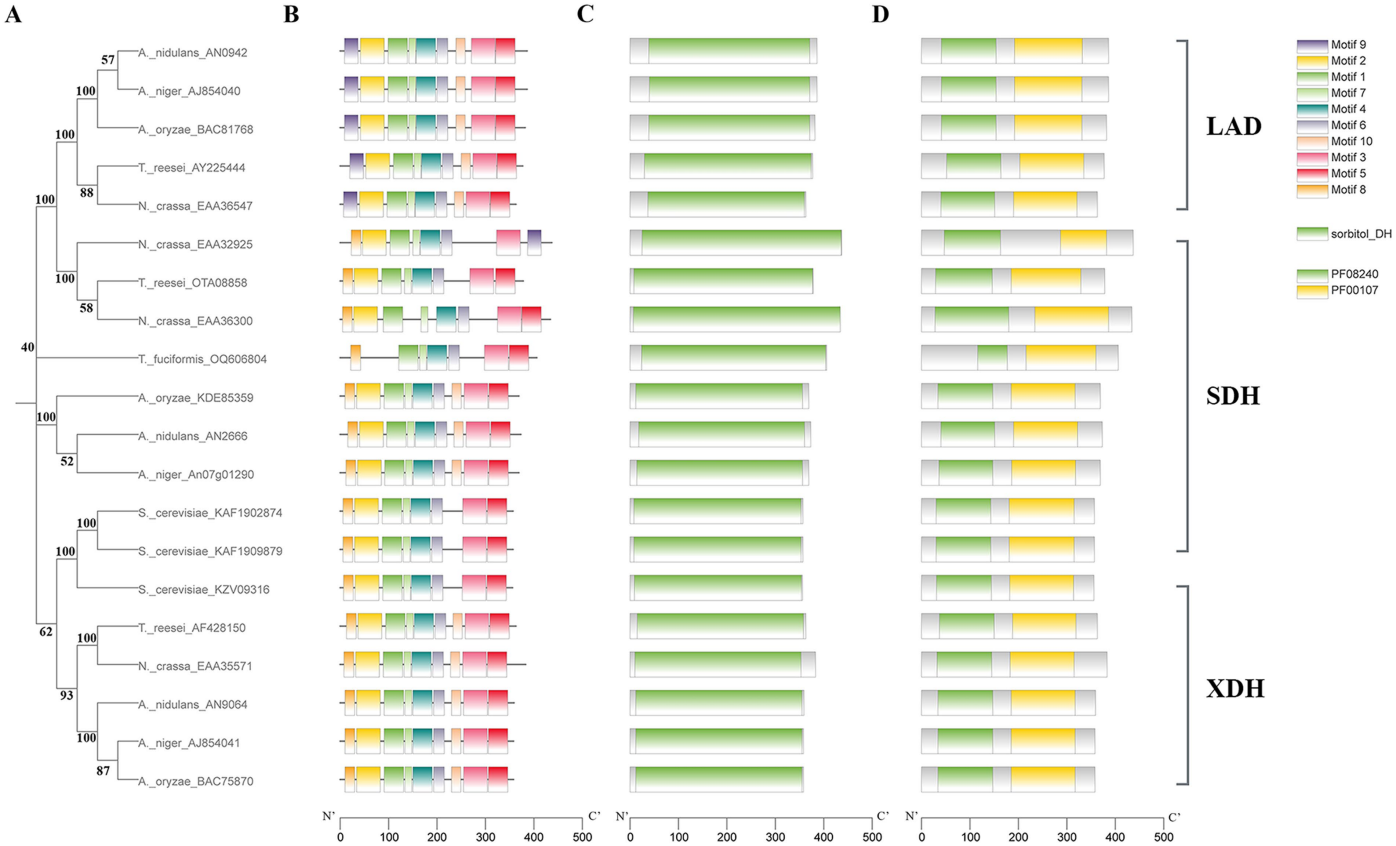
Bioinformatics analyses of TfSDH1 and L-arabitol, D-sorbitol, and xylitol dehydrogenases. **(A)** Neighbor-joining phylogenetic tree. **(B)** Identification of motifs. **(C)** Identification of protein family. **(D)** Identification of Pfam domains. The amino acid sequences of the respective proteins were retrieved either from GenBank, or translated from nucleotide sequences present in the respective genome databases. Organisms used were 5 ascomycete fungi: *A. niger*, *A. nidulans*, *A. oryzae*, *T. reesei*, *N. crassa*; 1 hemiascomycete fungus: *S. cerevisiae*; and 1 basidiomycete fungus: *T. fuciformis*.

The *Tfsdh1* gene (Genbank: OQ606804) comprises a 1,618-bp DNA sequence containing 6 typical class-II introns (5′GT-AG3′) of 56, 130, 52, 110, 76, and 60 nt, respectively. The coding sequence of *Tfsdh1* is 1,221 bp, encoding 406 amino acid residues. The molecular weight and theoretical isoelectric point of TfSDH1 were 43.03 kDa and 5.62, respectively. Signal peptide analysis predicted the absence of a signal peptide, indicating cytoplasmic localization of the protein. TfSDH1 shares sequence identities of 43.52% (*A. nidulans* AN2666), 42.86% (*A. niger* An07g01290), 41.69% (*A. oryzae* KDE85359), 37.82% (*T. reesei* OTA08858) and 34.84% (*N. crassa* EAA36300). In the neighbor-joining phylogenetic tree (Figure 5A), TfSDH1 forms an independent branch whereas shows closer phylogenetic proximity to SDH homologs, including *A. oryzae* KDE85359, *A. nidulans* AN2666, *A. niger* An07g01290, *T. reesei* OTA08858, and *N. crassa* EAA36300 and separated clearly from the LAD and XDH clades. Notably, *A. nidulans* AN2666 and *A. niger* An07g01290 have been experimentally confirmed as functional SDHs involved in the PCP and galactose metabolism (Koivistoinen et al., 2012; Meng et al., 2022), suggesting conserved catalytic roles for TfSDH1. MEME analysis (Figure 5B) indicated that motif 9 is present in all LADs but absent in all SDHs (except *N. crassa* EAA32925), its lack in TfSDH1 confirms its SDH classification. TfSDH1 was also the only sequence in the analyzed set missing motif 2. However, TfSDH1 contained motif 4, which corresponds to the conserved NAD^+^ cofactor-binding domain characterized by the sequence GXGXXG. This motif is essential for mediating interactions with the nucleotide moiety of NAD^+^, a critical requirement for the enzyme’s catalytic activity (Sellés Vidal et al., 2018). TfSDH1 belongs to the SDH-like family within the medium-chain dehydrogenase/reductase (MDR) superfamily (Figure 5C). TfSDH1 contains two conserved Pfam domains: PF08240 (Alcohol dehydrogenase GroES-like domain) and PF00107 (Zinc-binding dehydrogenase) (Figure 5D). The size of the PF08240 domain in TFSDH1 is highly shorter than other SDHs, suggesting potential functional significance.

### 3.3 *In silico* analysis of the *gpd* promoter

A 1,847-bp fragment containing the *gpd* promoter was obtained by PCR amplification. Analysis of this fragment using the NNPP identified 4 core promoter regions, of which transcriptional features including start/end positions, prediction scores, sequences, and initiation sites are detailed in Supplementary Figure S6A. Additionally, prediction of *cis*-regulatory elements revealed conserved motifs typical of eukaryotic promoters, such as the TATA-box, CAAT-box, and GC-motif (Supplementary Figure S6B). For vector construction, a truncated 503-bp fragment was selected that retains essential *cis*-elements while minimizing sequence length, ensuring functional integrity and transformation efficiency (Supplementary Figure S6B).

### 3.4 Generation and validation of transformants

To investigate the role of *Tfsdh1* in *T. fuciformis*, we generated *Tfsdh1* knockout and complementation strains. Primary screening by colony PCR confirmed successful transformation (Supplementary Figure S7B,E). Subsequent sequencing verified *Tfsdh1* deletion in the Y32 genome (Supplementary Figure S7D). qRT-PCR analysis demonstrated undetectable *Tfsdh1* expression in knockout strains (Supplementary Figure S7C), while complementation strains (com-*Tfsdh1* 1–5) exhibited 1 to 16-fold upregulation relative to wild type (WT) (Supplementary Figure S7F). These results collectively confirm *Tfsdh1* deletion and functional complementation.

### 3.5 Positive regulation of *Tfsdh1* for growth and morphology

**Figure 6:**
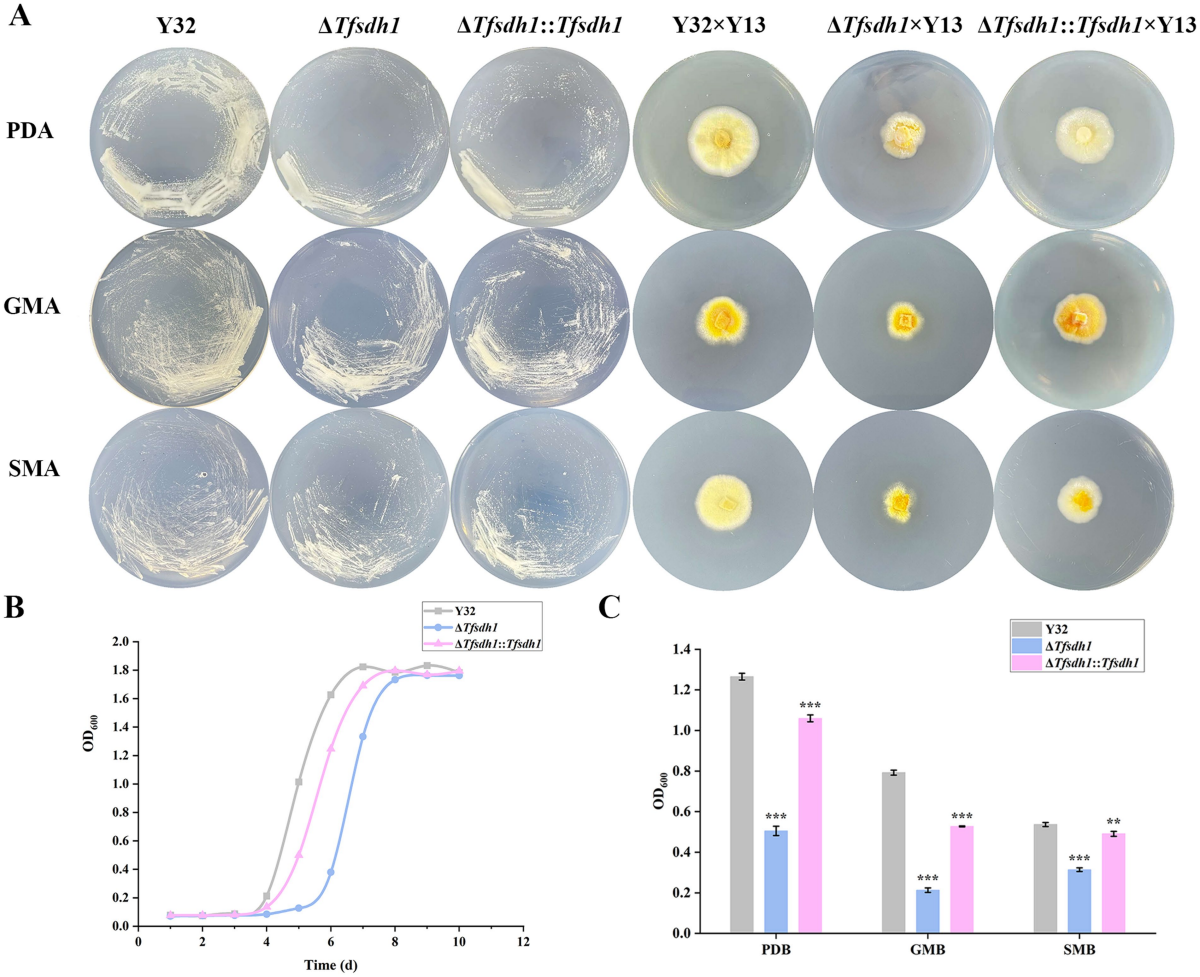
Phenotypic and growth analyses of different *T. fuciformis* strains under various conditions. **(A)** Monokaryotic and dikaryotic *T. fuciformis* strains cultured on PDA, GMA, and SMA. **(B)** Growth curves of *T. fuciformis* strains cultured in PDB. **(C)** OD_600_ values of *T. fuciformis* strains cultured in PDB, GMB, and SMB. Compared with WT strains, ***p < 0.001, **p < 0.01.

*Tfsdh1* significantly modulated growth dynamics and morphological characteristics of *T. fuciformis* across different culture conditions. When cultured on PDA, GMA, and SMA, Δ*Tfsdh1* and Δ*Tfsdh1* × Y13 exhibited reduced growth rates compared to complementation (com-*Tfsdh1* 1 and com-*Tfsdh1* 1 × Y13) and WT strains (Figure 6A). No morphological alterations were observed in monokaryotic strains under the same conditions (Figure 6A). However, on PDA plates, dikaryotic strains, Δ*Tfsdh1* × Y13 displayed suppressed hyphae formation, decreased mycelial density, and yellowish pigmentation around the colony center. These defective phenotypes were partially rescued in Δ*Tfsdh1*::*Tfsdh1* × Y13 (Figure 6A). On GMA and SMA, Δ*Tfsdh1* × Y13 displayed severely impaired radial growth, with the most pronounced growth retardation observed on SMA. Specifically, Δ*Tfsdh1* entered logarithmic phase 1–2 days later than Y32 and Δ*Tfsdh1*::*Tfsdh1* (Figure 6B). Extremely significant differences in growth rate were observed between Δ*Tfsdh1* and Y32 when cultured in PDB, GMB and SMB (*p* < 0.001, Figure 6C). It is worth mentioning that Δ*Tfsdh1* grew faster in SMB than in GMB, which was opposite of the growth pattern observed for Y32 and Δ*Tfsdh1*::*Tfsdh1* (Figure 6C).

### 3.6 Carbon source utilization analysis

**Figure 7:**
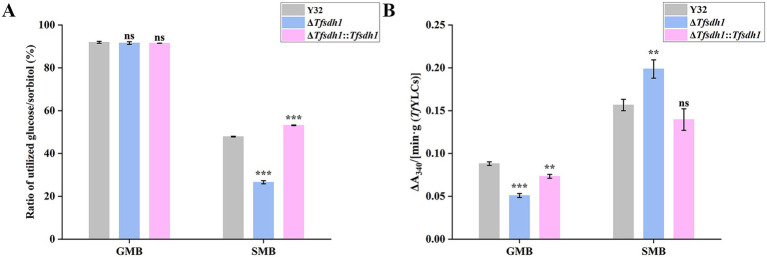
Carbon source utilization and enzymatic activity of different *T. fuciformis* strains. **(A)** Ratio of utilized glucose/sorbitol in *Tf*YLCs cultured in GMB and SMB. **(B)** Change in absorbance at 340 nm per minute per gram of *Tf*YLCs cultured in GMB and SMB. Compared with WT strains, ^***^p < 0.001, ^**^p < 0.01, ns represents no significance.

Notable differences in sorbitol catabolism were observed among *T. fuciformis* strains. After 5-day culture, glucose utilization rates of Y32, Δ*Tfsdh1*, and Δ*Tfsdh1*::*Tfsdh1* in GMB were 91.94, 91.60, and 91.49%, respectively (Figure 7A). No significant difference in glucose utilization rate were observed between Y32 and Δ*Tfsdh1*. In contrast, extremely differences in sorbitol utilization rate were detected when strains were cultured in SMB. Sorbitol utilization rates of Y32, Δ*Tfsdh1*, and Δ*Tfsdh1*::*Tfsdh1* were 47.88, 26.58, and 53.15%, accounting for 51.75, 29.18, and 57.53% of their respective glucose utilization rates. Compared to WT strains, the Δ*Tfsdh1* mutant exhibited 43.61% reduction (*p* < 0.001) in relative sorbitol utilization efficiency (Figure 7A).

### 3.7 TfSDH1 activity of *Tremella fuciformis* strains

The activity of TfSDH1 in *Tf*YLCs was differentially regulated in a carbon source-dependent manner (Figure 7B). When cultured in GMB, Δ*Tfsdh1* exhibited an extremely significant reduction in TfSDH1 activity, which was 42.27% lower than that of Y32 (*p* < 0.001) and 30.56% lower than that of Δ*Tfsdh1*::*Tfsdh1*. Notably, TfSDH1 activity in Δ*Tfsdh1*::*Tfsdh1* was restored to 83.13% of that in Y32. In contrast, when grown in SMB, Δ*Tfsdh1* showed a highly significant increase in TfSDH1 activity, with levels 26.91% higher than Y32 (*p* < 0.01) and 42.30% higher than Δ*Tfsdh1*::*Tfsdh1*, respectively. To explore the genetic basis for the increased activity, we analyzed the *T. fuciformis* genome for the presence of additional TfSDH1 homologs. A total of 13 genes encoding SDH were identified (see dataset_annotation_Tf in Supplementary material). Among these, 5 were differentially expressed, comprising 2 upregulated and 3 downregulated genes.

### 3.8 Interaction between *Tremella fuciformis* and *Annulohypoxylon stygium*

**Figure 8:**
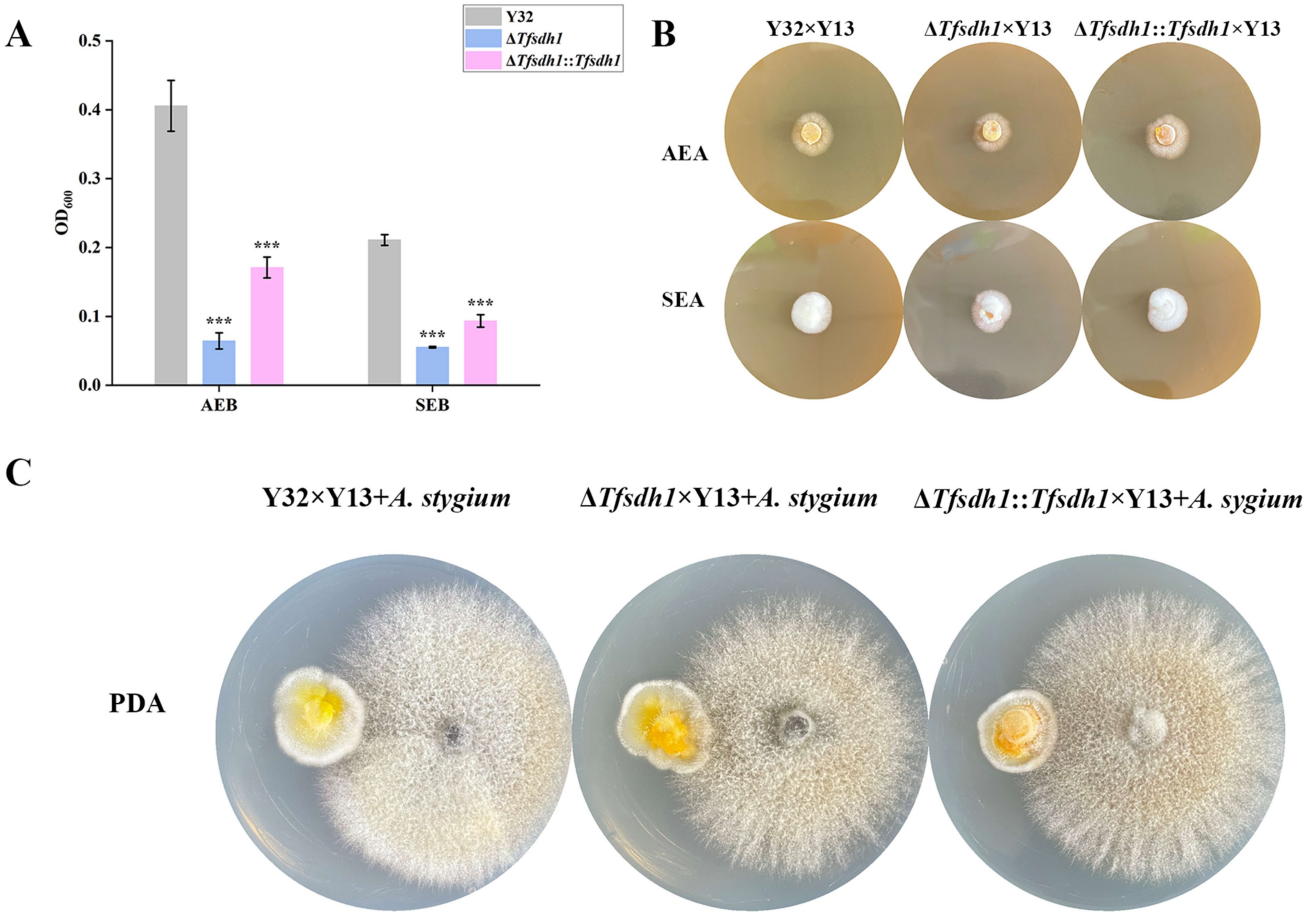
Interaction experiments of *T. fuciformis* and *A. stygium*. **(A)** OD_600_ values of *T. fuciformis* strains cultured in AEB and SEB. **(B)** Colony morphology of *T. fuciformis* mycelia cultured on AEA and SEA. **(C)** Interaction phenotype of *T. fuciformis* and *A. stygium* mycelia. Left colony on plate was *T. fuciformis* mycelia and right colony on plate was *A. sygium* mycelia.

The interaction between *T. fuciformis* and *A. stygium* was modulated by *Tfsdh1*. When cultured in AEB, Δ*Tfsdh1*::*Tfsdh1* exhibited faster growth than Δ*Tfsdh1*, but slower growth than WT strains. Notably, both Y32 and Δ*Tfsdh1*::*Tfsdh1* showed significantly accelerated growth in AEB compared to their growth in SEB, whereas Δ*Tfsdh1* displayed comparable growth rate in these two media (Figure 8A). Compared with SEA, gelatinization progress was delayed across all dikaryotic strains, including Y32 × Y13, Δ*Tfsdh1* × Y13, and Δ*Tfsdh1*::*Tfsdh1* × Y13, when cultured on AEA. In addition, although mycelia of all dikaryotic strains exhibited progressive whitening and increased densification, Δ*Tfsdh1* × Y13 maintained lower hyphal density than Y32 × Y13 and Δ*Tfsdh1*::*Tfsdh1* × Y13 (Figure 8B). This phenotypic difference was consistently observed in hyphal confrontation zones adjacent to *A. stygium* (Figure 8C). Collectively, these results suggest that extracts from *A. stygium* can stimulate the growth of *T. fuciformis*, and *Tfsdh1* plays a crucial role in mediating this interactive growth response.

## 4 Discussion

### 4.1 Sample preparation of interacted fungi

For comparative transcriptome analyses, interaction assays were conducted on wood substrates to simulate the natural growth environment of *T. fuciformis* and *A. stygium*. Since physically separating fungal mycelia from wood substrates was challenging, total RNA was extracted by grinding the mycelium-substrate mixture in liquid nitrogen, supplemented with an increased volume of TRIzol reagent, followed by standard procedures.

Ideally, samples A, B, and C should represent substrate-cultured mycelia. However, *T. fuciformis* cannot grow independently on wood substrates without its companion, *A. stygium*. Thus, sample A used *T. fuciformis* mycelia harvested from PDA culture. This approach likely introduces artifacts in interpreting gene expression patterns, particularly concerning lignocellulose degradation and starch and glucose metabolism. For instance, *T. fuciformis* mycelia on PDA likely express minimal levels of lignocellulose-degrading genes, and primary carbon sources in PDA stimulate the expression of starch and glucose metabolism-related genes, which differ significantly from those in natural wood substrates. While we recognize this limitation and the potential for misinterpretation, no superior alternative method was available at the time. We anticipate future studies will develop improved strategies to address this challenge.

### 4.2 Comparative transcriptome analysis

KEGG pathway enrichment analysis unveiled a fundamental divergence in the metabolic strategies of *T. fuciformis* and its companion fungus, *A. stygium*. Three significantly enriched pathways, including tyrosine metabolism, glutathione metabolism, and phenylalanine metabolism, were downregulated. Tyrosine and phenylalanine are primary precursors for various secondary metabolites, including melanins, lignans, and flavonoids (Feng et al., 2015; Esmaeilzadeh Bahabadi et al., 2012; Mohanta, 2020), while glutathione is central to antioxidant defense and detoxification (Wangsanut and Pongpom, 2022). This suggests that *T. fuciformis*, likely due to a stable or symbiotic relationship with *A. stygium*, may experience a reduced need for independent, high-intensity stress tolerance mechanisms, possibly relying on its partner for certain protective functions or environmental modification.

This hypothesis of metabolic division of labor is further supported by the analysis of the top-ranked upregulated pathways in *T. fuciformis*. Compared to *T. fuciformis*, *A. stygium* exhibits superior lignocellulose-degrading capacity. Degradation products such as D-glucose (from cellulose) and pentoses (from hemicellulose) significantly increase environmental carbon availability during their interaction. This likely activates starch and sucrose metabolism in *T. fuciformis* to decompose free glucose for energy. Concurrently, elevated pentose concentrations induce the pentose and glucuronate interconversion pathway, which synergizes with PCP, ORP, PPP, and glycolysis to enhance pentose utilization in *T. fuciformis*. Transcriptomic analysis confirmed that both fungi encode ORP and PPP genes, which are constitutively expressed during their interaction. Previous research confirmed that D-xylose induces the secretion of (hemi) cellulases in *A. niger* and upregulates the expression of hydrolase genes (*cbh1*, *egl2*, *bgl1*) in *Penicillium verruculosum* via the transcription factor XlnR (Mach-Aigner et al., 2012; Kislitsin et al., 2021; Chulkin et al., 2024). This supports the inference that abundant environmental pentoses similarly induce pentose-degrading gene expression in *T. fuciformis* and *A. stygium*. Notably, *A. stygium* exhibits significantly fewer upregulated genes involved in pentose metabolism than *T. fuciformis*, indicating minimal resource competition. Instead, *T. fuciformis* actively utilizes pentoses through transcriptional activation of relevant genes.

Valine, leucine, and isoleucine belong to the branched-chain amino acids (BCAAs), and their biosynthesis serve as critical components of proteins and secondary metabolites with multifaceted physiological roles. While leucine regulates mammalian metabolism through mTOR signaling (Dimou et al., 2022), in filamentous fungi, BCAAs modulate mycelial growth. Proteomic evidence shows *Cordyceps sinensis* synthesizes BCAAs to enhance membrane stability under hypoxic stress (Tang et al., 2025), whereas cadmium-exposed *Stropharia rugosoannulata* downregulates valine, leucine, and isoleucine biosynthesis, inhibiting mycelial growth (Dong et al., 2023). Thus, the upregulation of valine, leucine, and isoleucine biosynthesis in *T. fuciformis* likely promotes the rapid production of proteinogenic amino acids to support growth and development. The co-upregulated 2-oxocarboxylic acid pathway sits at a critical metabolic crossroads, connecting carbon metabolism with the biosynthesis of amino acids.

In stark contrast, the lack of significantly enriched pathways in *A. stygium* likely reflects the relatively diffuse nature of its transcriptional response, with DEGs distributed across multiple biological processes without strong concentration in specific pathways. However, its top upregulated pathways indicate it likely act as a pioneer and defender. The upregulation of various types of N-glycan biosynthesis is critical for the efficient secretion of a wide array of glycosylated wood-degrading enzymes (van Eerde et al., 2020; Zhang et al., 2024). Arginine biosynthesis serves as a nitrogen storage strategy in nitrogen-poor environment and provides precursors for polyamines, which are crucial for stress tolerance (Wang et al., 2025; Yao et al., 2025). Non-homologous end joining (NHEJ) points to a need for robust DNA repair. Finally, the upregulation of thiamine metabolism, a vital cofactor, and 2-oxocarboxylic acid metabolism fuels these energetically demanding processes (Tyibilika et al., 2024).

In conclusion, these two fungi illustrate a symbiotic division of labor. *A. stygium* acts as the pioneer and defender, upregulating pathways for environmental breakdown, nutrient storage, and cellular protection. Meanwhile, *T. fuciformis* adopts the role of beneficiary, downregulating its own defenses during interaction and activating a metabolic network dedicated to growth and development. This complementary relationship provides a plausible molecular foundation for their successful coexistence.

### 4.3 Analysis of putative PCP and ORP pathways

The principal pentoses derived from lignocellulose hydrolysis include L-arabinose, D-xylose and D-galactose (Rutten et al., 2009). In filamentous fungi, the PCP pathway mediates L-arabinose and D-xylose degradation, while the ORP pathway represents an important galactose metabolic route (Fekete et al., 2004). Integrating the expression profiles of DEGs from this study with established mechanisms in *A. niger*, *A. nidulans* and *T. reesei*, we proposed putative PCP and ORP pathways in *T. fuciformis*. L-arabinose reductase (LarA), which catalyzes the conversion of L-arabinose to L-arabitol in *A. niger* and *A. nidulans* (de Groot et al., 2005; Mojzita et al., 2010a; Flipphi et al., 2009; Meng et al., 2022), was not detected in either *T. fuciformis* or *A. stygium*. Instead, D-xylose reductases (XyrA, XyrB, XYL1) participate in L-arabinose reduction in *A. niger*, *A. nidulans*, and *T. reesei* (de Groot et al., 2005; Akel et al., 2009; Seiboth et al., 2007). These genes encoding D-xylose reductases are present in *A. stygium* genome but absent in *T. fuciformis* genome. During their interaction, *T. fuciformis* forms a functional haustorium, a specialized structure of parasitic fungi, through which *A. stygium* directly delivers nutrients (Zugmaier et al., 1994; Liu, 2010). In addition, sugar transporters from *A. stygium* facilitate the transfer of small-molecule sugars across a composite membrane formed between the two fungi (Lin, 2022). Based on these observations, we hypothesized that *A. stygium* supplies D-xylose reductase directly to *T. fuciformis* via the haustorium or composite membrane for L-arabitol production or *A. stygium* utilizes its own D-xylose reductase to reduce L-arabinose into L-arabitol, which is then transferred to *T. fuciformis* for further degradation by inducing the expression of corresponding enzymes in *T. fuciformis* during interaction. Notably, LarA, XyrA, and XyrB also participate in D-xylose reduction in *A. niger* and *A. nidulans* and XYL1 accounts for most of the reductase activity on D-xylose in *T. reesei* (Mojzita et al., 2010b; Seiboth and Metz, 2011). *A. stygium*-derived D-xylose reductase was considered for D-xylose reduction in *T. fuciformis* during the interaction. While *T. fuciformis* lacks the genes encoding L-arabinose reductase and D-xylose reductase, it possesses a gene encoding aldose reductase implicated in galactose oxidation in *A. nidulans* (Kowalczyk et al., 2015), which was undetected in *A. stygium*. Although D-xylose reductases also contribute to galactose reduction in *A. niger* and *T. reesei*, *A. stygium*-derived D-xylose reductase genes are non-DEGs, our putative ORP prioritizes *T. fuciformis*’s upregulated genes encoding aldose reductase.

Genes encoding L-arabitol dehydrogenase and D-xylitol dehydrogenase were detected in both *T. fuciformis* and *A. stygium*, whereas galactitol dehydrogenase homologs was absent in either species. In *A. niger* and *A. nidulans*, L-arabitol is converted to L-xylulose by L-arabitol dehydrogenase (LadA), D-xylitol dehydrogenase (XdhA), and sorbitol dehydrogenase (SdhA) (Chroumpi et al., 2022; Meng et al., 2022). In *T. reesei*, L-arabitol dehydrogenase (Lad1) and D-xylitol dehydrogenase (Xdh1) were confirmed to catalyze this reaction (Pail et al., 2004). These genes also participate in the conversion of xylitol to D-xylulose in *A. niger*, *A. nidulans* and *T. reesei*. Galactitol oxidation exhibits significant interspecific variability in model fungi. In *A. nidulans*, galactitol can be oxidized to L-sorbose by either galactitol dehydrogenase or L-arabitol dehydrogenase (Fekete et al., 2004; Christensen et al., 2011; Meng et al., 2022). In *A. niger*, it is oxidized to L-xylo-3-hexulose specifically by galactitol dehydrogenase (LadB). In *T. reesei*, this reaction is mediated by L-arabitol dehydrogenase (Lad1) from the PCP (Pail et al., 2004; Mojzita et al., 2012b). Given that *T. fuciformis* possesses L-arabitol dehydrogenase but lacks galactitol dehydrogenase, we speculated that the L-arabitol dehydrogenase in *T. fuciformis* may participate in galactitol oxidation. However, the exact product of this reaction remains unconfirmed, which could be L-sorbose, L-xylo-3-hexulose, or both, as observed in other fungal species. Downstream conversion of L-sorbose to D-sorbitol was catalyzed by L-xylulose reductase (LxrA) in *A. nidulans* (Seiboth and Metz, 2011). The reduction of L-xylo-3-hexulose to D-sorbitol is catalyzed by L-xylo-3-hexulose reductase (XhrA) in *A. niger* (Mojzita et al., 2012a). Finally, conversion of D-sorbitol to D-fructose is catalyzed by sorbitol dehydrogenase (SdhA) in *A. niger* and *A. nidulans* (Koivistoinen et al., 2012; Kowalczyk et al., 2015), whereas by a xylitol dehydrogenase in *T. reesei* (Mojzita et al., 2012a).

### 4.4 CRISPR/Cas9 system establishment in *Tremella fuciformis*

Gene knockout is employed to completely eliminate the function of targeted genes, allowing investigation of their biological importance through phenotypic analyses. To achieve this, the CRISPR/Cas9 system was adopted due to its high efficiency and precision in generating targeted double-strand breaks (DSBs), which significantly facilitates homologous recombination (HR)-mediated gene replacement (Vonk et al., 2019). In contrast, non-homologous end joining (NHEJ), rather than HR, is the predominant DSB repair pathway in filamentous fungi, which often leads to random integration and results in low knockout efficiency (van Peer et al., 2010; Ohm et al., 2010). Although disruption of NHEJ-related genes, such as *ku70*, *ku80*, and *lig4* can improve HR rates, such strategies may cause pleiotropic effects such as reduced antifungal resistance or impaired growth (Zhang et al., 2011; Gandía et al., 2016).

A major challenge in applying CRISPR/Cas9 to *T. fuciformis* was the uncertainty about Cas9 codon optimization and the lack of identified endogenous U6 promoters for sgRNA transcription. To circumvent these obstacles, an *in vitro* strategy described in *Schizophyllum commune* was employed here (Vonk et al., 2019). Cas9 protein was purified from *E. coli*, and sgRNAs were transcribed *in vitro* under the control of T7 promoter. These components were then co-transformed into protoplasts of *Tf*YLCs for gene editing. This *in vitro* sgRNA transcription approach is consistent with the strategy used in *Ganoderma lucidum* (Qin et al., 2017). Notably, this study represents the first successful application of CRISPR/Cas9-mediated gene editing in *T. fuciformis*, providing a valuable reference for genetic manipulation in mushrooms.

For the donor DNA construct, 1-kb homologous arms were used to balance HR efficiency and vector size, which minimizes potential negative effects on transformation. An endogenous promoter was employed to support gene expression in *T. fuciformis*. A dual-resistance selection system was implemented using the hygromycin resistance gene (*hph*) to identify HR-mediated knockouts, and the phleomycin resistance gene (*phleo*) to counter-select random NHEJ integration events (van Peer et al., 2009). This strategy significantly enhanced the screening of precise gene replacement mutants.

It should be noted that, in this study, gene knockout was achieved in monokaryotic *Tf*YLCs, which represents the first successful gene editing in this species. Due to technically demanding and time-consuming, it is challenged to generate homozygous knockouts in the dikaryotic mycelial stage. Therefore, mutant *Tf*YLCs were crossed with WT compatible mates to obtain dikaryotic mycelia. As a result, the mycelial phenotype reflects a heterozygous knockout background. Although this approach may not fully replicate the phenotype of a pure homozygous knockout, the pronounced phenotypic defects observed still provide valuable and reasonable inferences regarding gene function. This strategy offers a feasible and informative reference for future functional studies in basidiomycetes with similar genetic complexity.

### 4.5 Gene function of *Tfsdh1* during the interaction

Based on comparative transcriptome analysis, *Tfsdh1* was identified that was significantly upregulated during the interaction between *T. fuciformis* and *A. stygium*. Although its exact function requires further confirmation, current evidence suggests that *Tfsdh1* encodes a functional SDH in *T. fuciformis*, playing a central role in both sorbitol utilization and the interaction with *A. stygium*.

Bioinformatic analysis provided initial clues to the function of *Tfsdh1*. Both phylogenetic tree analysis and sequence feature identification support its classification within the SDH protein family. Although TfSDH1 forms an independent branch in the phylogenetic tree, this position is consistent with the deep phylogenetic divergence between the basidiomycete *T. fuciformis* and the ascomycete species, such as *Aspergillus*, *Trichoderma*, and *Neurospora*. Crucially, TfSDH1 is unequivocally nested within a major clade of partially confirmed ascomycete SDH homologs, strongly supporting its classification as a functional SDH that retains the core enzymatic activity. The disruption of Y318 residue is associated with higher affinity for D-sorbitol, which is commonly found in the protein sequence of LAD, structurally indicating that *Tfsdh1* is more likely to encode SDH or XDH rather than LAD (Rutten et al., 2009). Furthermore, this classification is supported by the conserved absence of motif 9 in TfSDH1 and other SDHs, a pattern that starkly contrasts with its universal presence in LADs. The exact functional role of motif 9 remains to be elucidated. However, well-documented substrate promiscuity exists between SDH and XDH, meaning both xylitol and sorbitol can be the substrates of SDH and XDH (Chroumpi et al., 2021; Meng et al., 2022). This characteristic makes definitive distinction based solely on bioinformatic analysis challenging. Consequently, the enzymatic kinetic properties (Km, Vmax, and Kcat) of recombinant protein is necessary for substrate determination. Unfortunately, recombinant expression in *E. coli* system yielded inactive inclusion bodies. Subsequent expression in yeast system with eukaryotic-specific post-translational modifications will be a crucial step for definitive functional characterization.

Despite the aforementioned uncertainties, *in vivo* and *in vitro* functional assays provide evidence supporting the core role of *Tfsdh1* in sorbitol metabolism. When cultured in media containing either glucose or sorbitol as the sole carbon source, *Tfsdh1* mutant strain displayed markedly reduced growth rates and abnormal hyphal morphology, with more pronounced defects on sorbitol. This phenotype differs from that in *A. niger* (Koivistoinen et al., 2012), where *sdhA* knockout mutant failed to grow on sorbitol but showed no impairment on glucose, implying a potentially broader role in metabolic regulation in *T. fuciformis*. Further carbon source utilization assays revealed a substantial decline in the capacity of mutants to decompose sorbitol, whereas glucose metabolism remained unaffected, supporting direct involvement of *Tfsdh1* in sorbitol degradation. Collectively, these findings indicate that *Tfsdh1* plays an essential role in sorbitol metabolism and impairs overall growth via indirectly interfering carbon regulatory networks or energy homeostasis. Enzyme activity assays revealed complex regulation. SDH activity drastically decreased in *Tfsdh1* mutant under glucose conditions, confirming that *Tfsdh1* is the major contributor to SDH activity. SDH activity was strongly induced in all strains when sorbitol served as the sole carbon source, suggesting sorbitol acts as an inducer regulating its expression. Surprisingly, *Tfsdh1* knockout mutant showed higher residual SDH activity than WT under sorbitol induction. A possible explanation could be that the presence of sorbitol induces compensatory expression from other functionally redundant genes. For example, in yeast, even when a key gene is knocked out, its paralogous genes can still maintain normal physiological function (Diss et al., 2017). Among the 13 SDH homologs identified, 5 showed differential expression, 2 of which were upregulated. *Tfsdh1*, the focus of this study, is one of these upregulated genes. After its knockout, the other upregulated SDH homologs may act as a functional substitute, thereby sustaining overall SDH activity in the absence of *Tfsdh1*. Furthermore, the deletion of *Tfsdh1* may trigger a feedback regulatory mechanism, leading to compensatory expression of other enzymes capable of utilizing sorbitol as a substrate, in an attempt to overcome the metabolic blockage. Similar compensatory regulatory mechanisms have also been reported in other fungi. For instance, in *Candida albicans*, amino acid starvation alters the activity of the central aromatic amino acid biosynthesis pathway (shikimate pathway). This induces the expression of two gene clusters involved in metabolizing hydroxybenzene derivatives, enabling the uptake and utilization of aromatic compounds from the environment (Garbe et al., 2025). In *A. oryzae*, deletion of the major facilitator superfamily (MFS) transporter *AoKat1*, which is responsible for transporting the industrially important secondary metabolite kojic acid, upregulates the transcription of another MFS transporter, *KojT* (Chen et al., 2025). In *Ganoderma lucidum*, silencing of the β-1,3-glucan transferase gene *gl20535* induces upregulation of its isozyme gene *gl24465* (Liu et al., 2025). These examples suggest that compensatory feedback may be a conserved mechanism in fungi in response to genetic perturbation. Notably, under sorbitol conditions, the *Tfsdh1* mutant exhibited increased SDH enzyme activity but a decreased rate of sorbitol utilization. The underlying mechanism is likely to be complex. We speculate that although compensatory expression enhances the activity of relevant intracellular enzymes, the gene knockout may simultaneously disrupt downstream metabolic processes, such as cofactor regeneration or post-translational regulation (Groth et al., 2022), thereby impeding normal enzyme function. The mutation may also impair the sorbitol transport system, limiting intracellular substrate availability and ultimately leading to the observed phenotype. The above speculations require further experimental validation.

The ultimate focus of this study was to elucidate the ecological role of *Tfsdh1* during the interaction. Our experiments confirmed that culture filtrate of *A. stygium* contains approximately 0.4 g/L of sorbitol (data not shown), providing a material basis for the growth. WT strains could efficiently utilize the sorbitol in the filtrate to growth, whereas *Tfsdh1* knockout mutant showed almost no growth in this medium. Similar results were observed in co-culture with *A. stygium*. These results revealed that *A. stygium*, as a decomposer, degrades complex carbon sources like lignocellulose into intermediate products readily absorbed by *T. fuciformis*. *T. fuciformis*, in turn, highly expresses relevant genes, including those for sorbitol metabolism, to utilize these intermediates, thereby driving its own rapid growth and development. This perfectly illustrates the division of labor and mutual benefit between these two fungi.

## Data Availability

The datasets presented in this study can be found in online repositories. The names of the repository/repositories and accession number(s) can be found in the article/Supplementary material.

## Footnotes

1
https://www.omicsmart.com/

2
http://bioinformatics.psb.ugent.be/webtools/plantcare/html/

3
http://chopchop.cbu.uib.no/
