# Supplementary material for: Comparative transcriptome analyses and CRISPR/Cas9-mediated functional study of Tfsdh1 reveal insights into the interaction between Tremella fuciformis and Annulohypoxylon stygium
Source: Front Microbiol. 2026 Jan 12;16:1723122. doi: 10.3389/fmicb.2025.1723122 (PMC12834513; doi:10.3389/fmicb.2025.1723122)
Supplement: Supplementary file 1 [file Data_Sheet_1.zip › Supplementary figures and tables.docx]

Supplementary Material

# Supplementary Figures and Tables

## Supplementary Figures


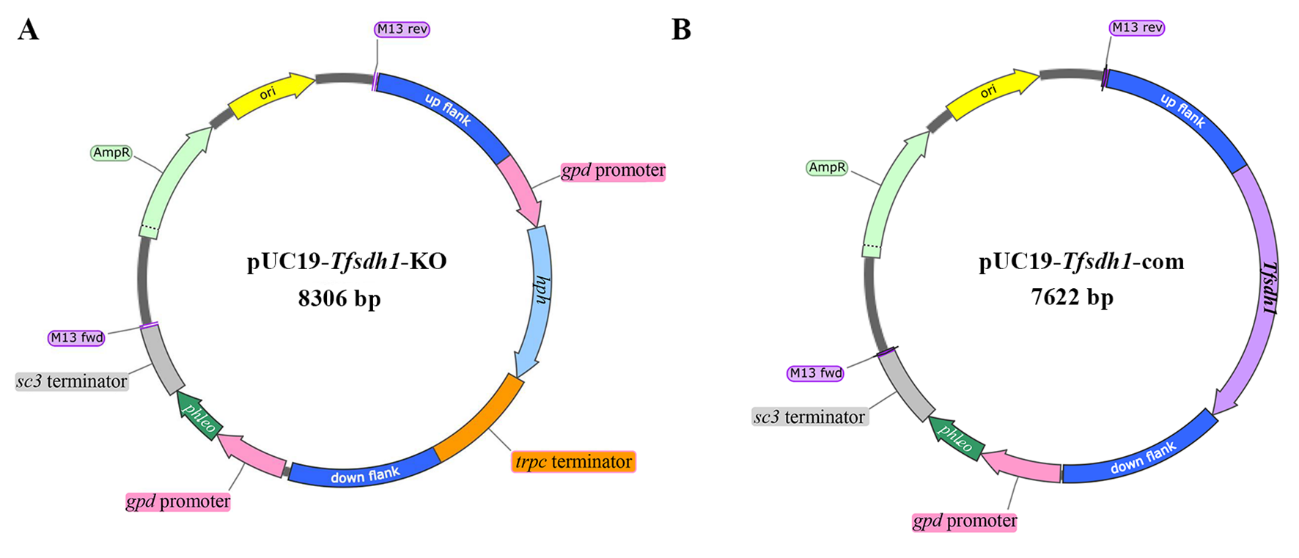


## Supplementary Figure S1. Vector graphs of pUC19-*Tfsdh1*-KO and pUC19-*Tfsdh1*-com.

##
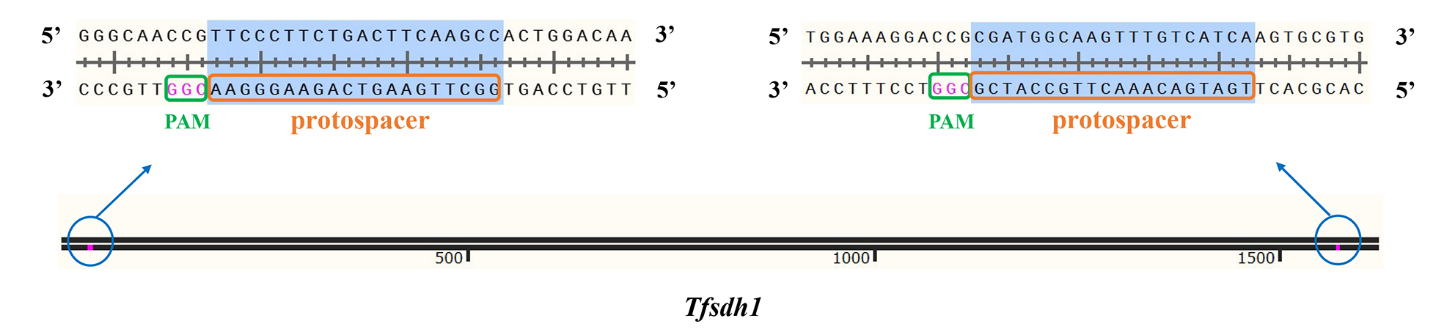


## Supplementary Figure S2. Schematic diagram of the protospacers in *Tfsdh1* gene.


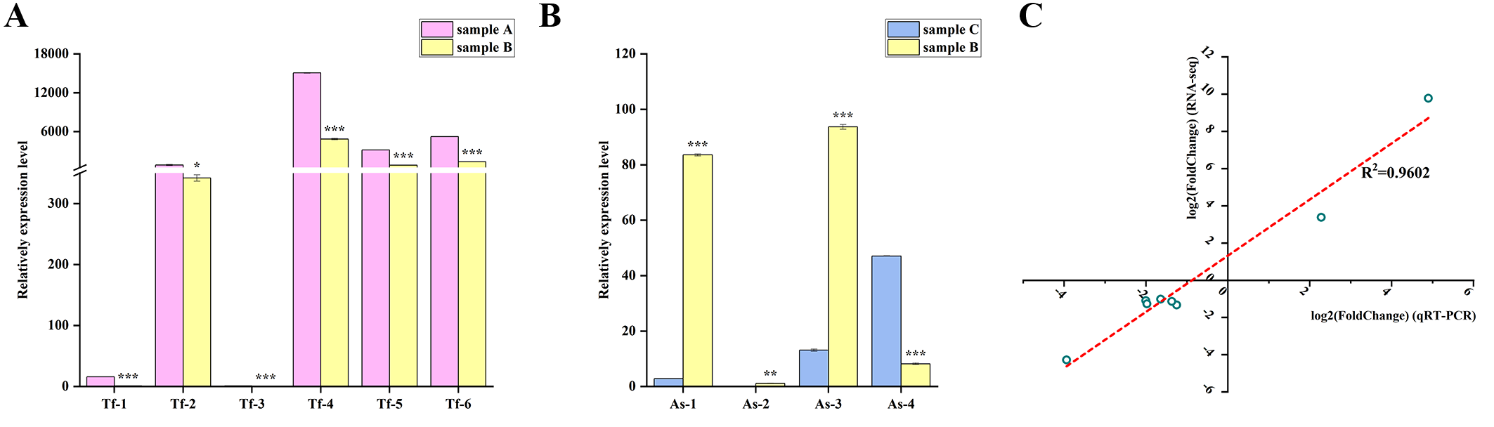


**Supplementary Figure S3.** Expression analysis of randomly selected DEGs in different samples. (A) Relative expression levels of genes in sample A and sample B. Tf-1, Tf-2, Tf-3, Tf-4, Tf-5, and Tf-6 were DEGs selected from *T*. *fuciformis*. Tf-1: gene_sp10029100.1, putative short chain dehydrogenase; Tf-2: gene_sp10019010.1, putative lactate dehydrogenase; Tf-3: gene_sp10058900.1, putative amidase; Tf-4: gene_sp10022810.1, putative cystathionine beta-synthase; Tf-5: gene_sp10022060.1, putative cytochrome c; Tf-6: gene_sp10056200.1, putative Mito-gen-activated protein kinase. (B) Relative expression levels of genes in sample C and sample B. As-1, As-2, As-3, and As-4 were DEGs selected from *A*. *stygium*. As-1: gene_sam0047700.1, putative acid phosphatase; As-2: gene_sam0062240.1, putative hypothetical protein; As-3: gene_sam0095580.1, putative luciferase-like monooxygenase; As-4: gene_sam0032390.1, putative SUZ domain. ****P*<0.001, ***P*< 0.01, **P*< 0.05, ns represents no significance. (C) Correlation analysis of qRT-PCR and RNA-seq data. The red dashed line represents the linear regression, with *R*²=0.9602 indicating a high correlation.


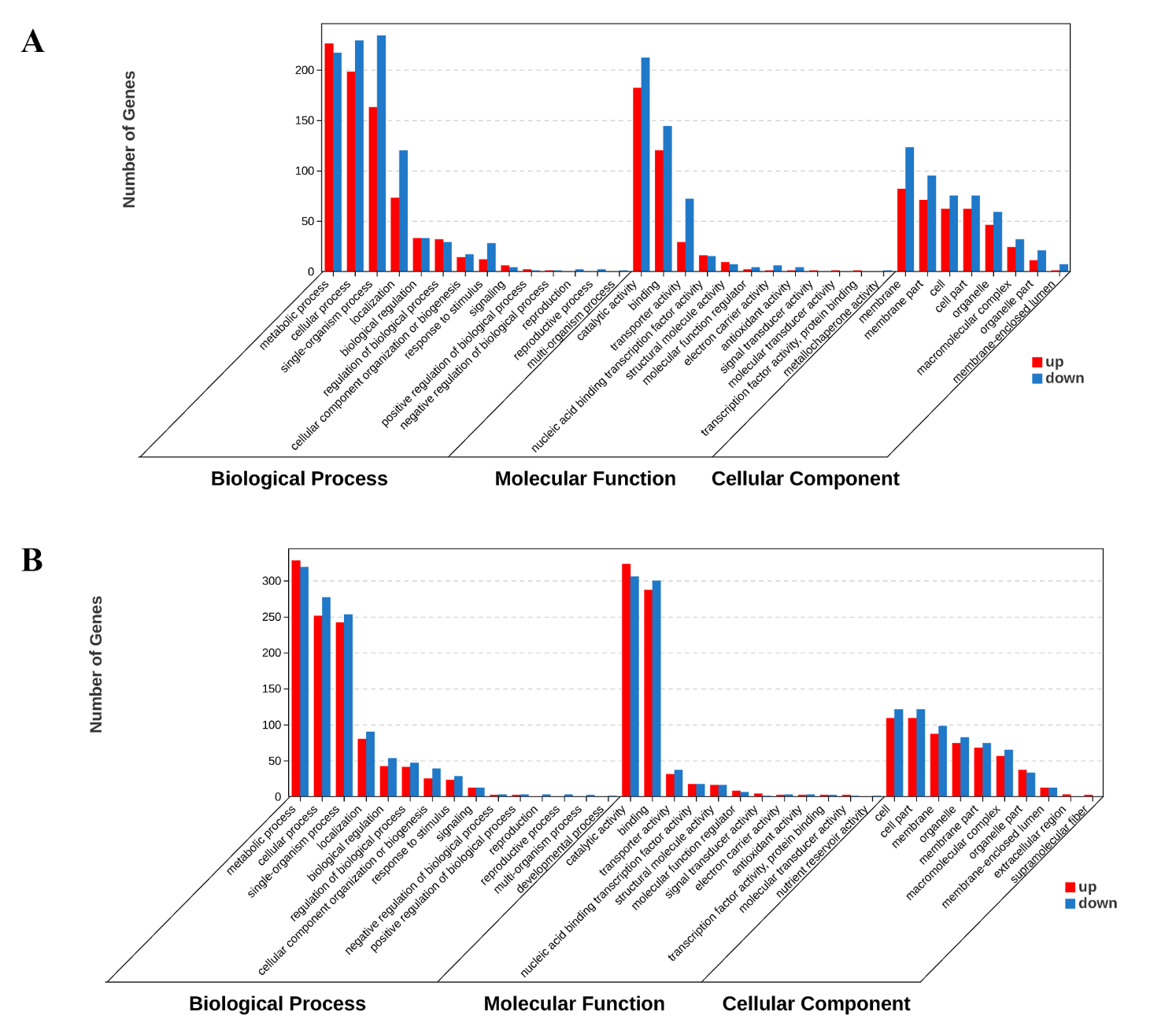


**Supplementary Figure S4.** GO annotation classifications of DEGs in *T*. *fuciformis* and *A*. *stygium*. (A) GO annotation classifications of DEGs in *T*. *fuciformis*. (B) GO annotation classifications of DEGs in *A*. *stygium*. X-axis represents GO terms and Y-axis represents number of genes. Red and blue columns represent up- and down-regulated genes in each GO term, respectively.


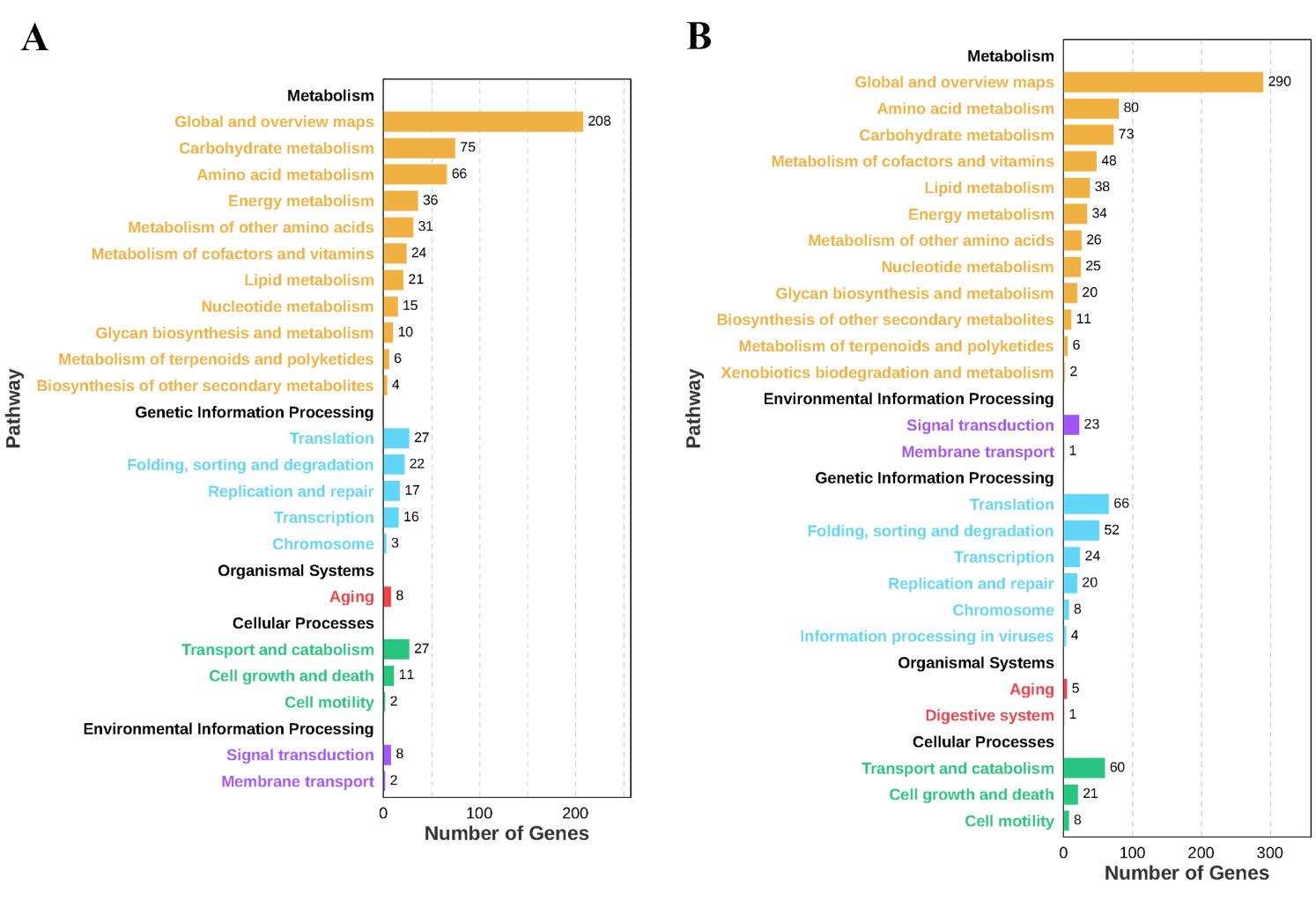


**Supplementary Figure S5.** KEGG annotation classifications of DEGs in *T*. *fuciformis* and *A*. *stygium*. (A) KEGG annotation classifications of DEGs in *T*. *fuciformis*. (B) KEGG annotation classifications of DEGs in *A*. *stygium*. X-axis represents number of genes and Y-axis represents KEGG pathways.


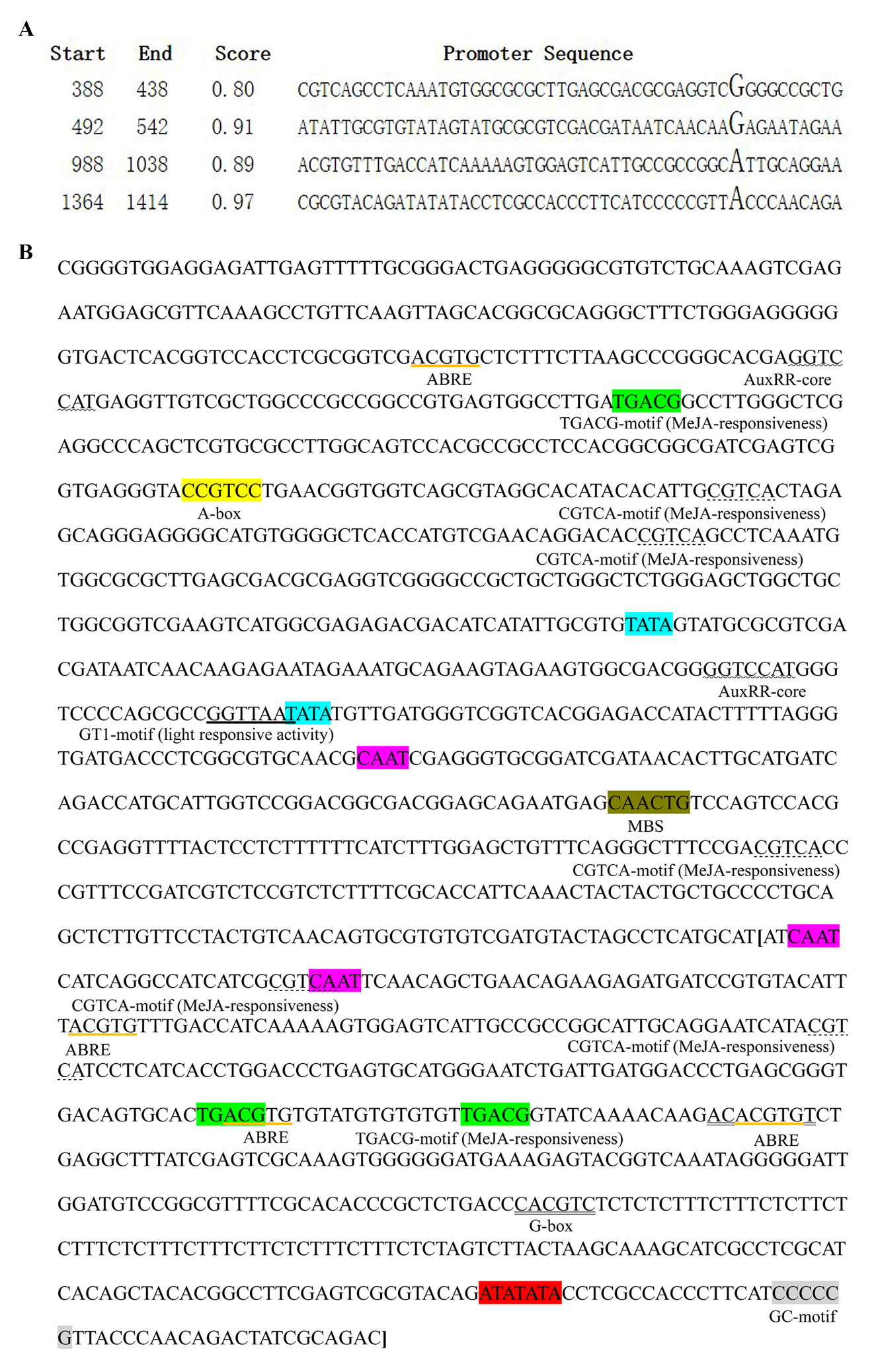


**Supplementary Figure S6.** Sequence analysis of *gpd* promoter from *T. fuciformis*. (A) Prediction of core *gpd* promoter regions from *T*. *fuciformis*. (B) *Cis*-elements of *gpd* promoter from *T*. *fuciformis*. Common *cis*-elements of promoter were marked in different colors or underlined. The 503 bp fragment inside parentheses was the shorter *gpd* promoter used in this study.


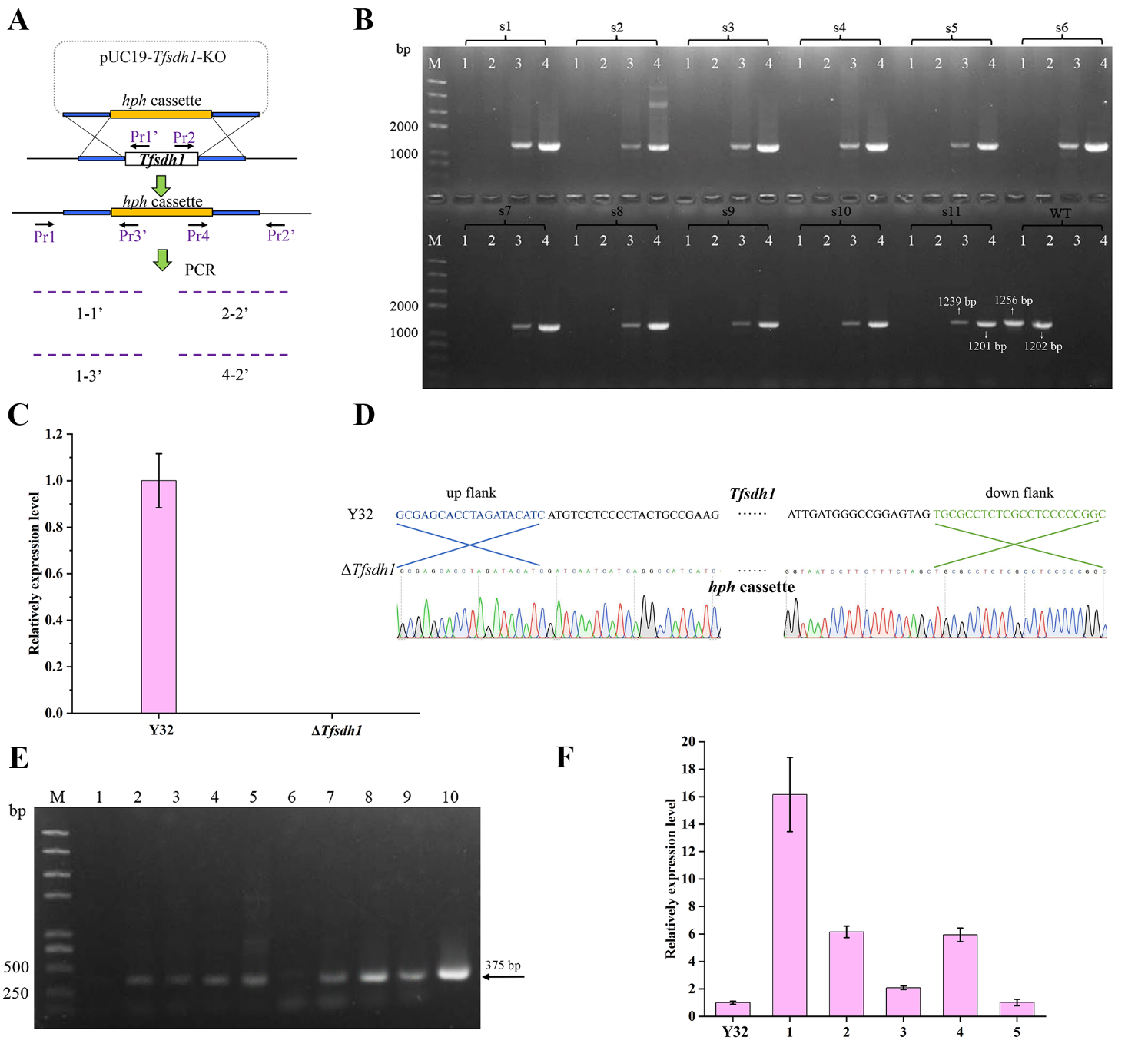


**Supplementary Figure S7.** Validation of transformants. (A) Schematic diagram of primers for Δ*Tfsdh1* screening. Pr1, Pr1’, Pr2, Pr2’, Pr3’ and Pr4 are primers. Pr1: *Tfsdh1*-chkA; Pr1’: *Tfsdh1*-chkB; Pr2: *Tfsdh1*-chkC; Pr2’: *Tfsdh1*-chkD; Pr3’: *hph*-chkB; Pr4: *hph*-chkC. (B) Colony PCR of putative *Tfsdh1* gene deletion transformants. Lane M: *Trans* 2K plusⅡmarker; Lane 1: using Pr1 and Pr1’ as the primers; Lane 2: using Pr2 and Pr2’ as the primers; Lane 3: using Pr1 and Pr3’ as the primers; Lane 4: using Pr4 and Pr2’ as the primers; s1-s11: putative *Tfsdh1* gene deletion transformants; WT: Y32. (C) qRT-PCR of *Tfsdh1* gene in Δ*Tfsdh1* and WT. (D) Sequencing verification of Δ*Tfsdh1*. *Tfsdh1* in Y32 genome was replaced with *hph* cassette via HR repair mechanism. (E) Colony PCR of putative *Tfsdh1* complementary transformants. Lane M: *Trans* 2K plusⅡmarker; Lane 1: blank control, using ddH_2_O as template; Lane 2-9: putative *Tfsdh1* complementary transformants; Lane 10: positive control, using pUC19-*Tfsdh1*-com as the template. (F) qRT-PCR of *Tfsdh1* gene in *Tfsdh1* complementary transformants and WT. 1, 2, 3, 4, and 5 were com-*Tfsdh1* 1-5.

## Supplementary Tables

**Table S1.** Primers for qRT-PCR of randomly selected genes from RNA-seq data.

| **Primer name** | **Sequence (5’‎→3’)** | **Purpose** |
| --- | --- | --- |
| Tf-1-F | CTCCACCATCGTCCTCACAG | qRT-PCR for gene Tf-1 (gene_sp10029100.1) |
| Tf-1-R | GCTGGATGTTGGGAAAGGGA |  |
| Tf-2-F | GAAGTCGCACAGCACAACAG | qRT-PCR for gene Tf-2 (gene_sp10019010.1) |
| Tf-2-R | TGGCATCGGGTGGATGTATG |  |
| Tf-3-F | GTGCGTTTCTCCCCGTAGAT | qRT-PCR for gene Tf-3 (gene_sp10058900.1) |
| Tf-3-R | GGCTTGAGCGAGTAGAGACC |  |
| Tf-4-F | CAACTACGTGATCGAGGGCA | qRT-PCR for gene Tf-4 (gene_sp10022810.1) |
| Tf-4-R | TTGGATTCTCAGCGACCACC |  |
| Tf-5-F | CGAGGACACCATGTTCGAGT | qRT-PCR for gene Tf-5 (gene_sp10022060.1) |
| Tf-5-R | GAGGTCGTTCCTGTCCTTGG |  |
| Tf-6-F | GCTTTCGGTCTCGTTTGCTC | qRT-PCR for gene Tf-6 (gene_sp10056200.1) |
| Tf-6-R | AGGTGAGAAGACGGTGCAAG |  |
| *β*-*tubulin*-F | AGGAGGAACTGGGTCTGGTCT | qRT-PCR for *β*-*tubulin* |
| *β*-*tubulin*-R | GACGACGGTTTCGGACACTT |  |
| As-1-F | AGCAAAGACGACTACGACGG | qRT-PCR for gene As-1 (gene_sam0047700.1) |
| As-1-R | GTTGCTGTCTGAGTGACCGA |  |
| As-2-F | CGTTCCGTTTGCTGTCAAGG | qRT-PCR for gene As-2 (gene_sam0062240.1) |
| As-2-R | GCCATAAGGAGAGCGAGTCC |  |
| As-3-F | TTTCTACCTGCACCACGGAC | qRT-PCR for gene As-3 (gene_sam0095580.1) |
| As-3-R | CACGAAGCAGAAAGGCGATG |  |
| As-4-F | ATCAGCCTCTCCCTCACCAT | qRT-PCR for gene As-4 (gene_sam0032390.1) |
| As-4-R | GTCTCTGGCACGAACATGGA |  |
| *α*-*tubulin*-F | TTGGAACGCCCTAACTATGAG | qRT-PCR for *α*-*tubulin* |
| *α*-*tubulin*-R | GAAGTGGATACGAGGATACGG |  |

*β*-*tubulin*: reference gene of *T*. *fuciformis*; *α*-*tubulin*: reference gene of *A*. *stygium*; gene_sp10029100.1: putative short chain dehydrogenase; gene_sp10019010.1: putative lactate dehydrogenase; gene_sp10058900.1: putative amidase; gene_sp10022810.1: putative cystathionine beta-synthase; gene_sp10022060.1: putative cytochrome c; gene_sp10056200.1: putative Mitogen-activated protein kinase; gene_sam0047700.1: putative acid phosphatase; gene_sam0062240.1: putative hypothetical protein; gene_sam0095580.1: putative luciferase-like monooxygenase; gene_sam0032390.1: putative SUZ domain.

**Table S2.** Other primers used in this study.

| **Primer name** | **Sequence (5’→3’)** | **Purpose** |
| --- | --- | --- |
| *Tfsdh1*-F | ATGTCCTCCCCTACTGCCGAAG | Cloning of cDNA and DNA sequences of *Tfsdh1* |
| *Tfsdh1*-R | CTACTCCGGCCCATCAATCACG |  |
| *gpd*-chk-F | CGGGGTGGAGGAGATTGAG | Cloning of upstream region and partial sequence of *gpd* |
| *gpd*-chk-R | AGATTTGTGAAGGAACAGCGGAC |  |
| chkF | CTTTATGCTTCCGGCTCG | Colony PCR validation of pUC19-*sdh*-KO |
| chkR | GATGATGGCCTGATGATTGAT |  |
| *Tfsdh1*-chkA | GGCATGTAGGTGCAGCATC | Validation of *Tfsdh1* knockout transformants |
| *Tfsdh1*-chkB | CCTTTGTCCGCTCCTGAGT |  |
| *Tfsdh1*-chkC | ACCTGAAGCCCCTCTTGAC |  |
| *Tfsdh1*-chkD | GCCAACTCCATGTGCTGTC |  |
| *hph*-chkB | TGATTCCTGCAATGCCGGC |  |
| *hph*-chkC | CCATGGTTGCCTAGTGAATG |  |
| q*Tfsdh1*-F | ATCACGATCAAGGGCGGATG | qRT-PCR for *Tfsdh1* |
| q*Tfsdh1*-R | CGAGCGCGTCTGTAAACTTG |  |
| *phleo*-chk-F | ATGGCCAAGTTGACCAGTGC | Validation of *Tfsdh1* complementation transformants |
| *phleo*-chk-R | TCAGTCCTGCTCCTCGGCC |  |

**Table S3.** Primers used for vector construction.

| **Primer name** | **Sequence (5’→3’)** | **Purpose** | **Note** |
| --- | --- | --- | --- |
| p*gpd*-1-F | CTCGAGATCAATCATCAGGCCATCATC | Cloning of native *gpd* promoter for overlapped PCR | *Xho* I restriction enzyme site was underlined |
| p*gpd-*1-R | GCTTTTTCATGTCTGCGATAGTCTGTTGGGTAACGGGGGATGAAG |  |  |
| *hph*-F | TATCGCAGACATGAAAAAGCCTGAACTCACCGCGACGTCTGTCG | Cloning of *hph* and *trpc* terminator for overlapped PCR | *Xho* I restriction enzyme site was underlined |
| *hph*-R | CTCGAGCTAGAAAGAAGGATTACCTC |  |  |
| p*gpd*-2-F | AGAGGATCCCCGGGTACCGAGCTCGATCAATCATCAGGCCATCATC | Cloning of native *gpd* promoter with 25 bp overhang |  |
| p*gpd*-2-R | GTGATGGGCATGTCTGCGATAGTCTGTTGGGTAACGGGGGATGAA |  |  |
| *phleo*-F | TATCGCAGACATGCCCATCACCAACATGGCCAAGTTGACCAGTGC | Cloning of *phleo* and *sc3* terminator with 25 bp overhang |  |
| *phleo*-R | CGACGTTGTAAAACGACGGCCAGTGACGCGCCGAATTCCCAGCTTG |  |  |
| *Tfsdh1*-up-1-F | AACAGCTATGACCATGATTACGCCACTCGATCACACTACAGCAC | Cloning of *Tfsdh1* up flank with 25 bp overhang |  |
| *Tfsdh1*-up-1-R | CGCGATGATGGCCTGATGATTGATCGATGTATCTAGGTGCTCGC |  |  |
| *Tfsdh1*-down-1-F | TTTAGAGGTAATCCTTCTTTCTAGCTGCGCCTCTCGCCTCCCCCG | Cloning of *Tfsdh1* down flank with 25 bp overhang |  |
| *Tfsdh1*-down-1-R | TCTAGAGTCGACCTGCAGGCATGCATGGCTCAGCAGGTGCATCAG |  |  |
| *Tfsdh1*-up-2-F | AACAGCTATGACCATGATTACGCCACTCGATCACACTACAGCAC | Cloning of fragment from *Tfsdh1* up flank to down flank |  |
| *Tfsdh1*-down-2-R | TGATTGATCGAGCTCGGTACCCGGGTGGCTCAGCAGGTGCATCAG |  |  |

**Table S4.** Primers used for sgRNA synthesis.

| **Primer name** | **Sequence (5’→3’)** | **Purpose** | **Note** |
| --- | --- | --- | --- |
| *Tfsdh1*-sgRNA-left-F | TAATACGACTCACTATAGGGCTTGAAGTCAGAAGGGAA | Synthesis of left sgRNA template | Left sgRNA is sgRNA close to initiation codons |
| *Tfsdh1*-sgRNA-left-R | TTCTAGCTCTAAAACTTCCCTTCTGACTTCAAGCC |  |  |
| *Tfsdh1*-sgRNA-right-F | TAATACGACTCACTATAGTGATGACAAACTTGCCATCG | Synthesis of right sgRNA template | Right sgRNA is sgRNA close to stop codons |
| *Tfsdh1*-sgRNA-right-R | TTCTAGCTCTAAAACCGATGGCAAGTTTGTCATCA |  |  |
| Tracr-F | GTTTTAGAGCTAGAAATAGCAAGTTAAAATAAGGCTAGTCCGTTATCAACTTGAAAAAGTGGCACCGAGTCGGTGCTTTT | Synthesis of sgRNA template |  |
| Tracr-R | AAAAGCACCGACTCGGTGCCACTTTTTCAAGTTGATAACGGACTAGCCTTATTTTAACTTGCTATTTCTAGCTCTAAAAC |  |  |
| Uni-F | TAATACGACTCACTATAG | Synthesis of sgRNA template |  |
| Uni-R | AAAAGCACCGACTCGGTGCCAC |  |  |
